# Tissue clearing of both hard and soft tissue organs with the PEGASOS method

**DOI:** 10.1038/s41422-018-0049-z

**Published:** 2018-05-29

**Authors:** Dian Jing, Shiwen Zhang, Wenjing Luo, Xiaofei Gao, Yi Men, Chi Ma, Xiaohua Liu, Yating Yi, Abhijit Bugde, Bo O. Zhou, Zhihe Zhao, Quan Yuan, Jian Q. Feng, Liang Gao, Woo-Ping Ge, Hu Zhao

**Affiliations:** 1 Department of Restorative Sciences, School of Dentistry, Texas A&M University, Dallas, TX 75246 USA; 2 State Key Laboratory of Oral Diseases, National Clinical Research Center for Oral Diseases, West China Hospital of Stomatology, Sichuan University, Chengdu, 610041 China; 3 Children’s Research Institute, Departments of Pediatrics, Neuroscience, Neurology and Neurotherapeutics, UT Southwestern Medical Center, Dallas, TX 75390 USA; 4 Live Cell Imaging Core Facility, UT Southwestern Medical Center, Dallas, TX 75390 USA; 5 State Key Laboratory of Cell Biology, Shanghai Institute of Biochemistry and Cell Biology, Chinese Academy of Sciences, University of Chinese Academy of Sciences, 320 Yueyang Road, Shanghai, 200031 China; 6 Intelligent Imaging Innovations (3i) Inc., 3509 Ringsby Court, Denver, CO 80216 USA

## Abstract

Tissue clearing technique enables visualization of opaque organs and tissues in 3-dimensions (3-D) by turning tissue transparent. Current tissue clearing methods are restricted by limited types of tissues that can be cleared with each individual protocol, which inevitably led to the presence of blind-spots within whole body or body parts imaging. Hard tissues including bones and teeth are still the most difficult organs to be cleared. In addition, loss of endogenous fluorescence remains a major concern for solvent-based clearing methods. Here, we developed a polyethylene glycol (PEG)-associated solvent system (PEGASOS), which rendered nearly all types of tissues transparent and preserved endogenous fluorescence. Bones and teeth could be turned nearly invisible after clearing. The PEGASOS method turned the whole adult mouse body transparent and we were able to image an adult mouse head composed of bones, teeth, brain, muscles, and other tissues with no blind areas. Hard tissue transparency enabled us to reconstruct intact mandible, teeth, femur, or knee joint in 3-D. In addition, we managed to image intact mouse brain at sub-cellular resolution and to trace individual neurons and axons over a long distance. We also visualized dorsal root ganglions directly through vertebrae. Finally, we revealed the distribution pattern of neural network in 3-D within the marrow space of long bone. These results suggest that the PEGASOS method is a useful tool for general biomedical research.

## Introduction

Tissue opaqueness is mainly derived from heterogeneous optical properties among different components. Water has refractive index (RI) of 1.33, proteins have RI of above 1.44 and lipids have RI of above 1.45.^1–3^ Mismatched RI among different components scatters the incoming light. In addition, endogenous pigments including heme, lipofuscin, and melanin block the light from transmission. Calcified mineral and collagen further block the light transmission in bone and dental tissues. All current tissue clearing techniques achieve transparency through similar physical principles, despite different chemical reagents being used. Transparency can be achieved through eliminating RI mismatch within the tissue and decolorizing pigment elements.^1,2^ The first tissue clearing technique was introduced by Werner Spalteholz over a century ago to study the tissue organization within the whole animal body.^4,5^ In recent years, many new tissue clearing methods were developed, including 3DISCO, FluoClear, uDISCO, Scale, SeeDB, CLARITY, CUBIC, PACT, SWITCH, CUBIC-R, and Bone Clarity et al.^6–19^ Current tissue clearing methods can be classified into two major categories based on the components of clearing medium: organic solvent-based methods and aqueous reagent-based methods. Organic solvent-based approaches obtain high tissue transparency by using clearing medium with high RI (RI >1.50). Most of the aqueous reagent-based methods have lower RIs (RIs < 1.49) and are more amenable for fluorescent protein.

Transparency, fluorescence preservation and tissue applicability are the three major criteria for evaluating a clearing method. Although whole-body imaging has been demonstrated in previous studies, all current clearing methods have limitation on types of tissues they can clear.^1,9,16,20^ Aqueous reagent-based clearing methods including CLARITY, PACT, and CUBIC-R efficiently cleared soft tissue, but not hard tissue organs.^1,16,20^ uDISCO was not efficient on clearing highly colorized organs including liver and spleen, and achieved only partial success on clearing hard tissue.^9^ Bone CLARITY was recently developed for clearing bones, but its clearing effects on soft tissue organs were not demonstrated.^15^ These limitations of the above-mentioned methods inevitably led to the presence of blind areas within a whole-body imaging.

Hard tissues make up over 15% of total body weight and are especially difficult to be cleared. Clearing of teeth, the hardest tissue in the body, has never been demonstrated by any previous methods. PACT and CUBIC could clear very thin calvarial bones but not long bones.^16,20^ uDISCO could clear bisected long bones.^9^ Bone CLARITY was specifically designed to clear long bones, but the entire clearing process takes around 1 month and the reagents were expensive.^15^

Organic solvent-based clearing methods usually achieved better transparency than aqueous reagent-based methods, but suffered from significant fluorescence loss. For example, GFP fluorescence level in samples decreased by over 50% 1 month after the treatment of uDISCO BABBD clearing medium.^9^ The fluorescence quenching was mainly attributed to low pH value, protein denaturation, and presence of free radicles within the solvent.^9,17^

Due to the above challenges, it is still imperative to develop a more general clearing technique applicable for diverse tissues with improved transparency while preserving endogenous fluorescence. Therefore, we designed the polyethylene glycol (PEG) associated solvent system (PEGASOS) tissue clearing method. We demonstrated that the PEGASOS method renders nearly all types of tissues transparent except pigmented epithelium. Hard tissues including bones and teeth become nearly invisible after clearing. Polyethylene glycol component within the clearing medium provided protection for endogenous fluorescence for a long time. With the PEGASOS method, we were able to image intact mouse head composed of bones, teeth, brain, muscles, and other tissue types. We also imaged intact mouse brain and traced individual axons and neurons over a long distance. We were able to image through the vertebrae to identify connections between the peripheral nervous system (PNS) and the central nervous system (CNS). Importantly, we also revealed the nerve distribution pattern within the marrow space of long bone.

## Results

### The PEGASOS recirculation method achieves whole-body transparency and enables large body part imaging without blind spots

**Fig. 1:**
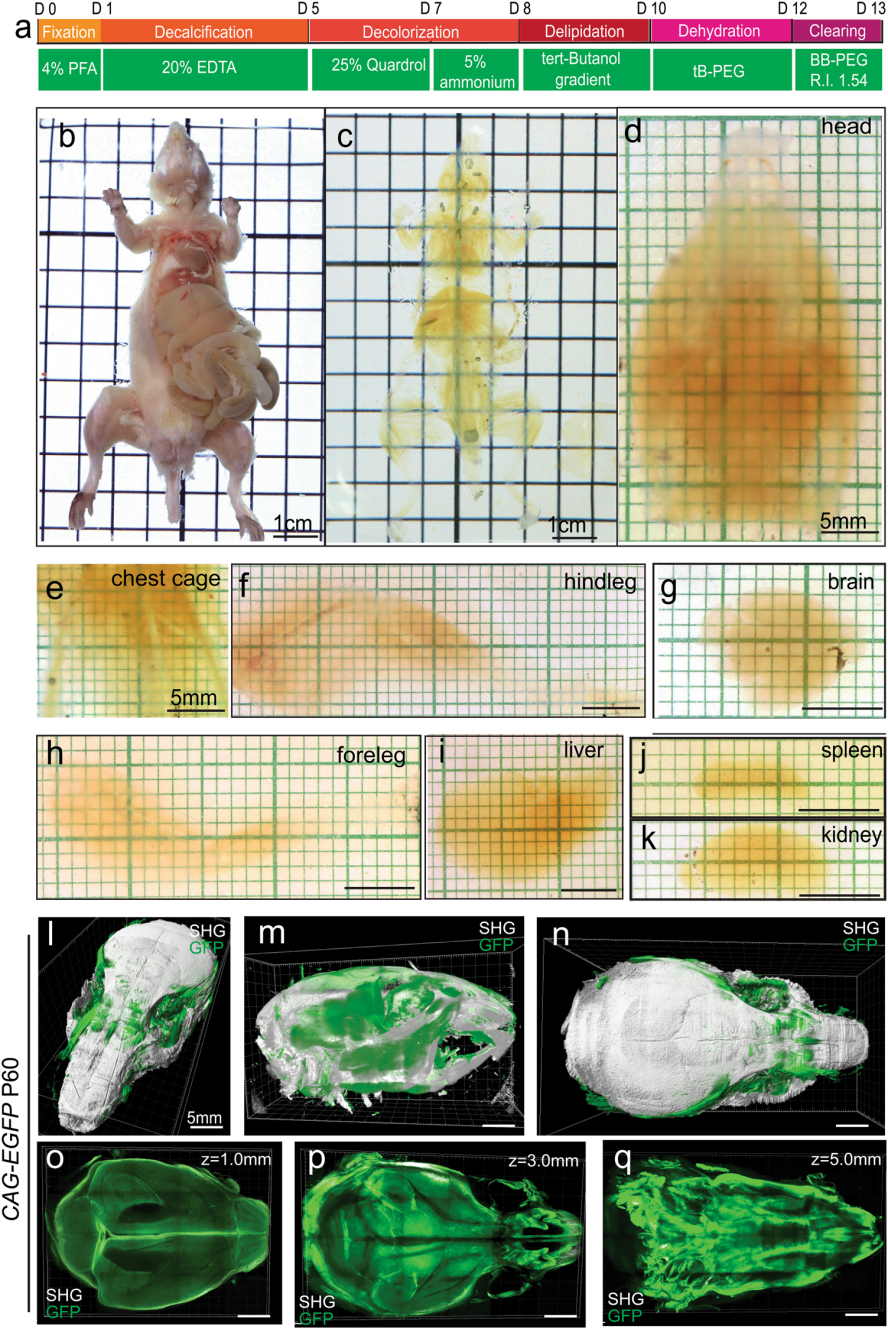
PEGASOS recirculation procedure achieves whole-body transparency and enables whole-head imaging with no blind area. **a** Brief description of the PEGASOS recirculation procedure. **b**–**c** Adult *CAG-EGFP* mouse (2-months old) was imaged before (**b**) and after (**c**) the recirculation procedure. The skin, eyeballs and tongue were removed to facilitate penetration. Other tissues including brain, bones, teeth, muscles, and glands remained intact. **d**–**k** Body parts and internal organs were dissected after clearing. **l**–**q** After clearing, the whole head was imaged with a two-photon microscope in both ventral-to-dorsal (V–D) and dorsal-to-ventral (D–V) directions. The two image stacks were stitched together with Image J. **o**–**q** Show optical sections acquired at different depths in the D–V direction. SHG second harmonic generation signal. Scale bars in **d**–**q**, 5 mm

The PEGASOS method is consisted of multiple steps including fixation, decalcification (hard tissue only), decolorization, delipidation, dehydration, and clearing (Fig. 1a). It can be performed in two approaches, the recirculation procedure for clearing the whole body or large body parts and the passive immersion procedure for clearing individual organs or small body parts. In the recirculation procedure, chemical reagents were infused through a perfusion needle left in the left ventricle to access the entire body. After 4% PFA fixation, 20% ethylenediaminetetraacetic acid (EDTA) solution was first perfused to decalcify the hard tissue. Next, we adopted 25% *N*,*N*,*N*′,*N*′-Tetrakis(2-Hydroxypropyl)ethylenediamine (Quadrol) in H_2_O solution as the decolorizing reagent.^1^ Ammonium solution was widely used for dissolving heme and for whitening bone samples in taxonomy.^21,22^ Therefore, we decided to combine 25% Quadrol and 5% ammonium solutions in sequence for decolorizing samples (Fig. 1a).

Methanol was used for dehydration in the iDISCO method.^23,24^ Tetrahydofuran (THF) was used for dehydration in the 3DISCO method.^10,25^ Both of them may cause severe quenching of GFP fluorescence. Tert-butanol (tB) supplemented with vitamin E was used in the uDISCO for dehydration, which provides better protection for GFP fluorescence.^9^ We applied tB supplemented with 3% Quadrol for delipidation. The Quadrol component functions to maintain the solution at pH 9.0. We also designed tB-PEG reagent for dehydration, which is composed of 75% tB + 22% poly(ethylene glycol) methacrylate (PEGMMA) + 3% Quadrol. For final tissue clearing, we designed BB-PEG medium (RI 1.543), which is composed of 75% benzyl benzoate (BB), 22% PEGMMA, and 3% Quadrol.

We attempted to clear the whole body of adult mice (postnatal day 60, P60). After perfusing the mice with 4% PFA, all the organs were kept except the skin, eyeballs and tongue being removed (Fig. 1b). A typical recirculation clearing procedure took 12 days (Fig. 1a). Images were acquired after each treatment step (Supplementary Information, Figure S1a–h). After clearing, the entire mouse body turned transparent (Fig. 1c, Supplementary Information, Figure S1i). The grids in the background can be clearly visualized through the body. The size of the body also shrunk significantly (Fig. 1c, Supplementary Information, Figure S1i). Body parts including the entire head (containing the brain), thorax, and both front and hind legs were highly transparent, suggesting successful clearing of all tissues on them. Complete transparency was also obtained for internal organs including the brain, liver, kidney, and spleen (Fig. 1d–k).

The recirculation procedure achieved high transparency of large body parts, which enabled us to image an adult mouse head composed of bones, muscles, brain, and other tissues. The cleared head of a *CAG-EGFP* mouse was imaged with a 5×/0.16 objective on a two-photon microscope. Second harmonic generation (SHG) fluorescence was used to show collagen-rich tissues such as bones and teeth. GFP fluorescence was used for imaging soft tissue because hard tissue contains fewer cellular components and displays weaker GFP fluorescence. The head (~10 mm in dorsal-to-ventral direction) was imaged in both the dorsal-to-ventral (6 mm Z-Stack) and ventral-to-dorsal (4 mm Z-stack) dimensions. The two stacks were stitched together with Image J to form a complete image stack (Fig. 1l, m, n). Optical sections obtained at 1, 3, and 5 mm depths revealed internal structures (Fig. 1o, p, q).

To investigate if neuronal tissue can be visualized through the bone, adult *Thy1-YFP-H* mice (P60) were cleared following the recirculation procedure. The skull containing the brain was isolated and imaged with a 5×/0.16 objective on a two-photon microscope. The head was imaged in dorsal-to-ventral direction (6 mm Z-stack). Brain organization was clearly demonstrated by imaging through the calvarial bone (Supplementary Information, Figure S2a, b). Optical sections acquired at X-Z directions revealed large anatomical structures, but at a low resolution due to the poor axial resolution of the 5×/0.16 objectives (Supplementary Information, Figure S2c, d). Much better resolution was achieved for X-Y optical slices and individual neurons could be visualized on sections acquired at 2, 3, and 5 mm Z-depths (Supplementary Information, Figure S2f, f’, g, g’, i). Cranial nerves exiting in the cranial cavity and nerves innervating facial muscles were also visualized (Supplementary Information, Figure S2g, h, h’). Facial muscle tissues show increased auto-fluorescence (Supplementary Information, Figure S2h’).

### PEGASOS passive immersion method efficiently clears nearly all types of tissues

**Fig. 2:**
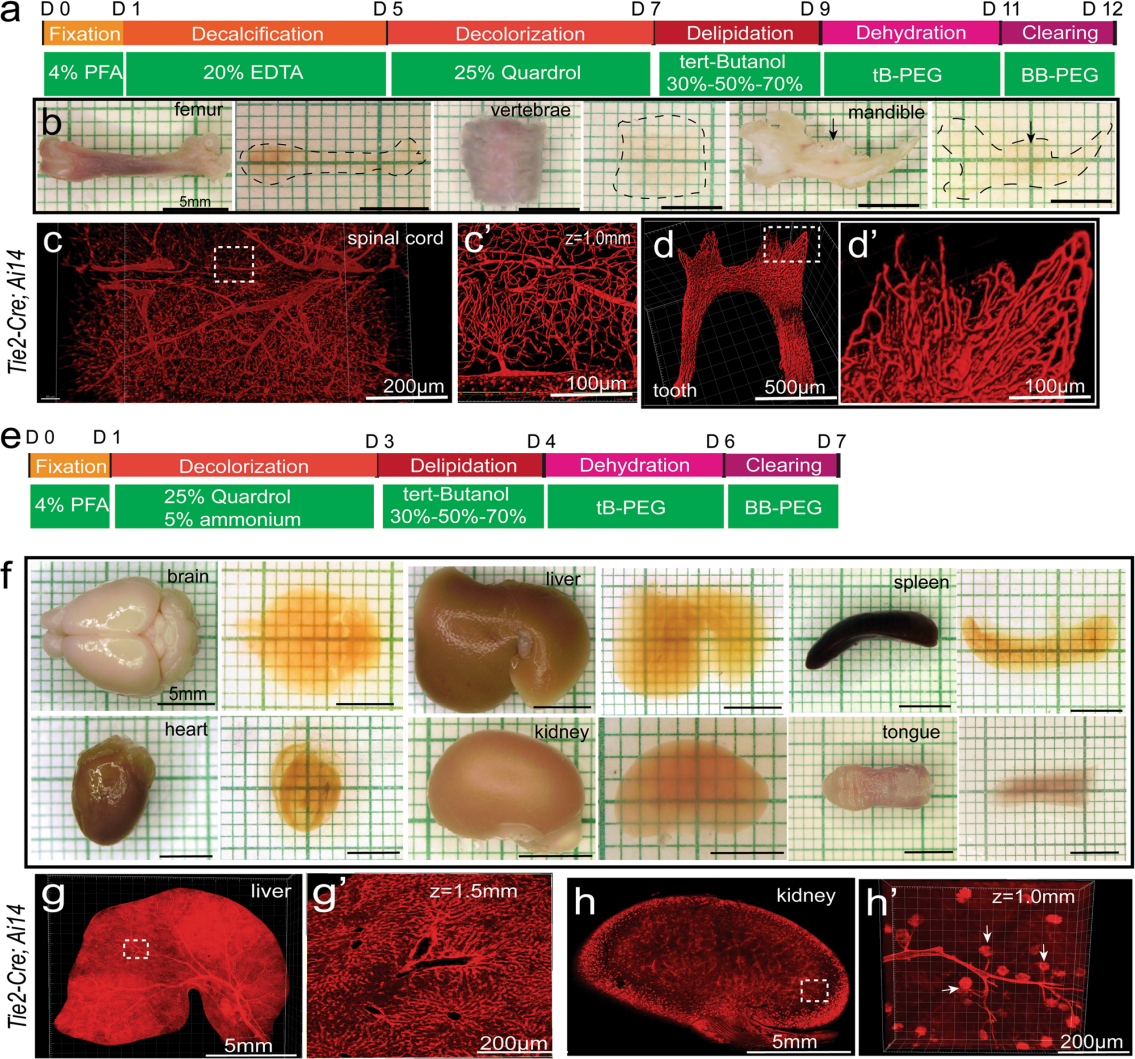
PEGASOS passive immersion procedure clears both hard and soft tissue organs. **a** Brief description of the PEGASOS passive immersion procedure for clearing hard tissue organs. **b** Femurs, short vertebrae segment ( < 3 cm length) and mandible were harvested from adult mice (60 days of age) and imaged before and after clearing. Dotted lines outline the organs after clearing. Arrows indicate teeth before and after clearing. **c** Short vertebrae from *Tie2-Cre; Ai14* mice was imaged through the bone to reveal the inside vasculatures. Boxed area is enlarged in **c’**. **d** Tooth of *Tie2-Cre; Ai14* was imaged after clearing to reveal the vascular network within the dental pulp. Boxed area is enlarged in **d’**. **e** Brief description of the PEGASOS passive immersion procedure for clearing soft tissue organs. **f** Various soft tissue organs harvested from adult mice (60 days of age) were imaged before and after clearing. An intact liver lobe (**g**) and an intact kidney (**h**) harvested from adult *Tie2-Cre; Ai14* mice (60 days of age) were imaged after clearing to reveal the vascular organization. Boxed areas are enlarged in **g’** and **h’**, respectively. Arrows in **h’** indicate renal capsules. Scale bars in **b** and **f**, 5 mm

The PEGASOS method can also be performed following the passive immersion procedure. For hard tissue, ~12 days were needed for final clearing (Fig. 2a). We were able to make intact femur, short vertebrae segment (less than 3 cm length), and mandible together with teeth nearly invisible (Fig. 2b). We imaged the vertebrae segment of *Tie2-Cre;Ai14* mouse with a confocal microscope and visualized the vasculature network within the spinal cord (Fig. 2c, c’). Tooth enamel and dentin are the hardest tissues in the body.^26^ By imaging the teeth on a cleared mandible of *Tie2-Cre;Ai14* mouse, we were able to visualize the enriched vascular network encapsulated by enamel and dentin (Fig. 2d, d’).

The PEGASOS passive immersion procedure could clear soft tissue organs in a much shorter time because decalcification is not needed. Within 7 days, we were able to make the brain, liver lobe, spleen, heart, kidney, stomach, intestine, and lung completely transparent (Fig. 2f; Supplementary Information, Figure S3a, a’, c’ c’, e, e’). We imaged and reconstituted the liver lobe and kidney harvested from *Tie2-Cre;Ai14* mice with a confocal microscope. Optical sections obtained at various levels revealed the vasculature organization within the liver and kidney (Fig. 2g, g’, h, h’). We imaged and reconstituted the intact stomach and intestine harvested from *Tie2-Cre;Ai14* mice and optical sections revealed the vasculatures in both organs (Supplementary Information, Figure S3b, b’, d, d’). We imaged and reconstituted the right lung from a *CAG-EGFP* mouse. Optical sections reveal the bronchi organization (Supplementary Information, Figure S3f, f’).

**Fig. 3:**
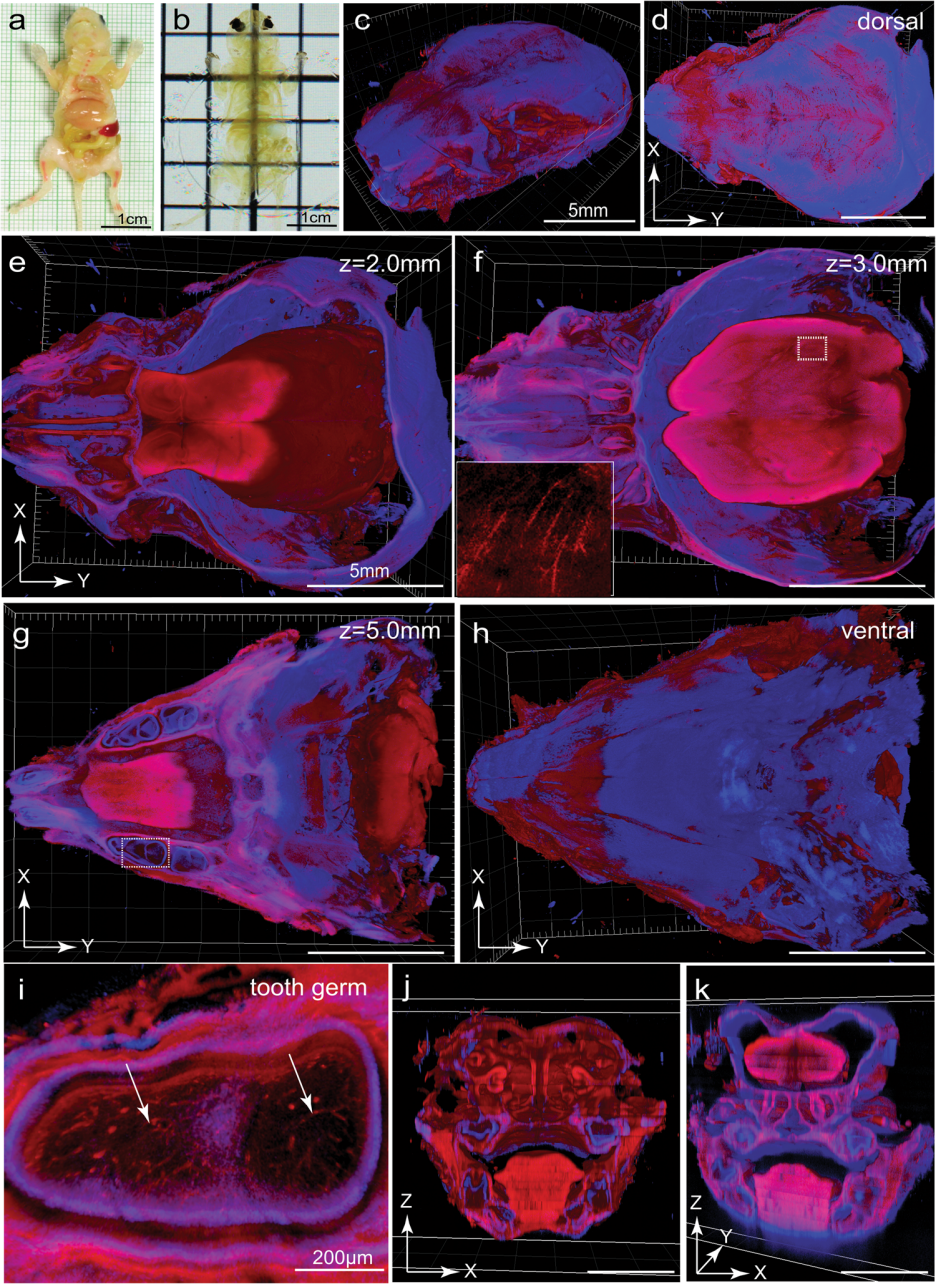
PEGASOS passive immersion procedure achieves whole-body transparency of mouse pups and enables whole-head imaging with no blind area. **a**, **b** Mouse pups of P7 age were cleared following the passive immersion procedure. **c** A *Tie2-Cre;Ai14* mouse pup (P7) after clearing was imaged with a 5× objective on a two-photon microscope. SHG signal was displayed in blue. TdTomato signal was displayed in red. Space between the brain and calvarial bone was caused by brain shrinkage. **d**–**k** Optical sections at different Z-depths, including the dorsal surface (**d**), 2.0 mm (**e**), 3.0 mm (**f**), 5.0 mm (**g**), and the ventral surface (**h**), were acquired. Dotted box in **f** was enlarged in the insert to show blood vessels. Dotted box in **g** was enlarged in **i** to show blood vessels within the tooth germ (arrows). X-Z optical slices acquired at different locations were displayed in **j**, **k**. SHG, second harmonic generation signal. Scale bars in **c**–**h**, **j** and **k**, 5 mm

Although adult full body clearing requires the recirculation procedure, whole body of young mouse pups could be cleared following the passive immersion procedure for the hard tissue due to their smaller size. A *Tie2-Cre;Ai14* mouse pup of postnatal day 7 age (P7) was cleared and showed full transparency 12 days after fixation except the pigmented eyes (Fig. 3a, b). The head containing the brain was removed for imaging with a 5×/0.16 objective in both the dorsal-to-ventral (5 mm Z-stack depth) and ventral-to-dorsal directions (1 mm Z-stack depth). After stitching the two stacks together, 3-D reconstitution showed intact head organization with both SHG and tdTomato signals (Fig. 3c, d, Supplementary Information, video 1). The brain separated from the calvarial bone due to shrinkage (Fig. 3e). X-Y optical sections obtained at 2, 3, and 5 mm in Z-depth revealed not only the large anatomical structures but also fine vasculatures (Fig. 3f). Vasculatures within the mandibular tooth germ were also clearly visualized (Fig. 3g, i). Large anatomical structures could be visualized on coronal optical sections acquired at X-Z directions, but with a lower resolution (Fig. 3j, k).

In summary, the PEGASOS method efficiently cleared all types of tissues except pigment retina.

### PEGASOS method provides a novel approach for investigating hard tissue organs in 3-D

**Fig. 4:**
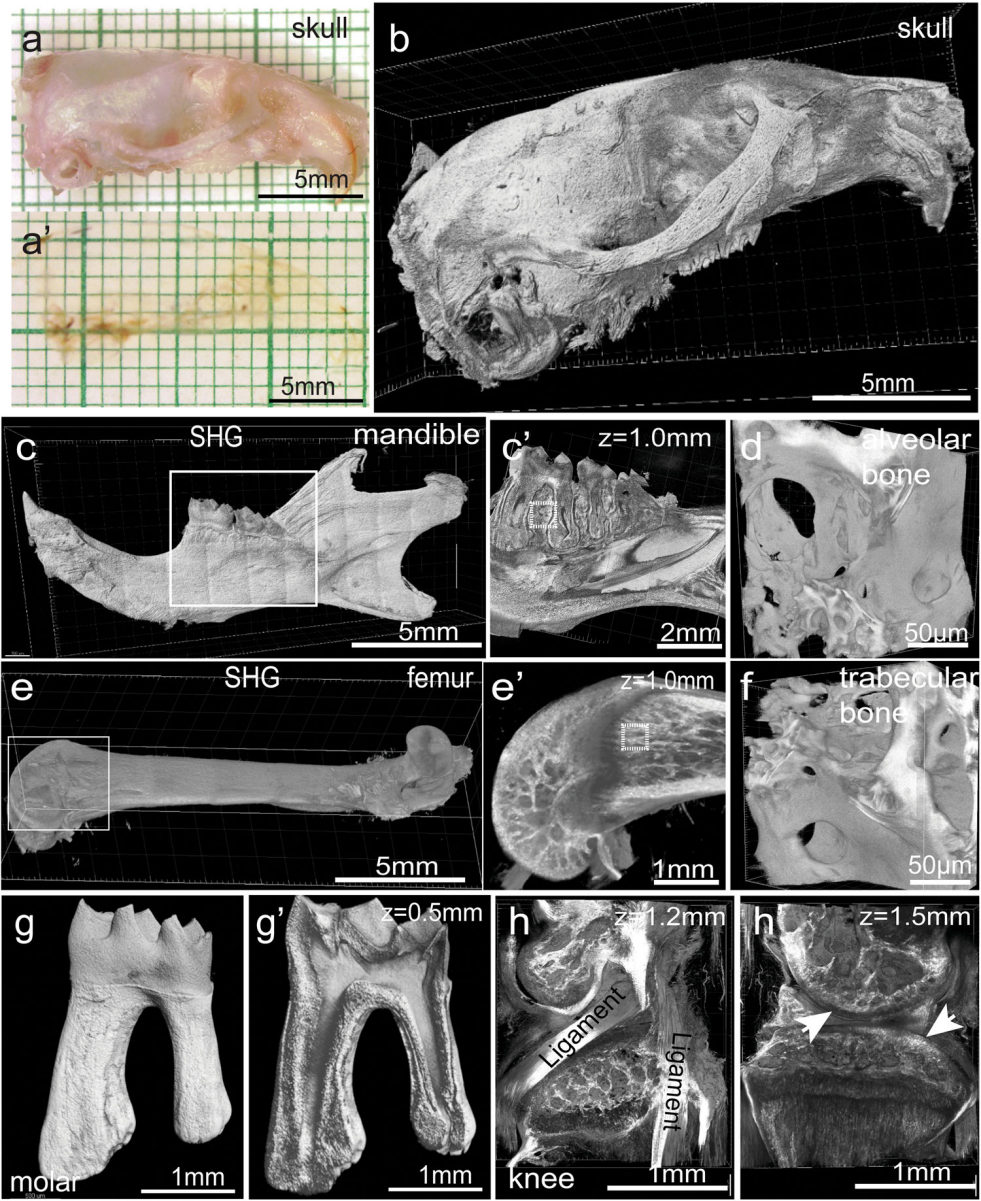
The PEGASOS method provides an approach for investigating hard tissue in 3-D. Hard tissue samples were harvested from adult mice and processed following the passive immersion procedure for hard tissue organs. Half of the skull was imaged with a stereomicroscope before (**a**) and after clearing (**a’**). **b** The cleared skull bone was imaged based on the SHG signal acquired with a 5× objective on a two-photon microscope. The intact mandible (**c**) and femur (**e**) were imaged. Optical sections were acquired at boxed regions and enlarged in **c’** and **e’** respectively. Boxed areas in **c’** and **e’** were re-imaged with a 32× 0.85 NA objective to reveal the trabecular bone organization of the alveolar bone (**d**) or femur (**f**). **g** Tooth within the mandible was imaged with a two-photon microscope. Optical section was obtained to show the pulp chamber (**g’**). **h** and **h’** An intact knee joint was cleared. Optical sections acquired at different depth are displayed. Arrows indicate the articular surface. Articular ligaments are labeled. SHG second harmonic generation signal

Complete transparency enables us to image the intact hard tissues including bones and teeth in 3-D with regular confocal or two-photon microscope. Based on the SHG fluorescent signal, we were able to reconstitute hard tissue samples in 3-D. We cleared half of the skull bone and imaged it with a 5×/0.16 objective on a two-photon microscope (Fig. 4a, a’, b). We reconstituted the entire mandible with a 10×/0.30 objective on a two-photon microscope. Optical sections showed the tooth structure within the mandible (Fig. 4c, c’, Supplementary Information, video 2). We reconstituted the entire femur with a 10×/0.3 objective and optical sections revealed the trabecular bone structure within the femur (Fig. 4e, e’, Supplementary Information, video 3). Alveolar bone and femur were re-imaged with a 20×/0.5 objective to reveal the spongy-like trabecular bone organization (Fig. 4d, f). The first molar on the mandible was re-imaged with a 20×/0.5 objective to achieve better resolution and details (Fig. 4g, g’). We reconstituted the knee joint and optical sections were obtained at different levels to show the articular surfaces and ligaments (Fig. 4h, h’).

### Performance comparison between the PEGASOS and other clearing methods

We compared the PEGASOS method with three other commonly used clearing methods on their clearing performance.^9,15,16^ Bone CLARITY was recently published for clearing hard tissues.^15^ Impressive transparency was achieved for adult mouse femur, mandible, and vertebrae, which is consistent with published results.^15^ However, Bone CLARITY was not effective for soft tissue organs including brain, liver, and spleen (Supplementary Information, Figure S4a2 to f2**)**. CUBIC-R method yielded excellent clearing outcome for soft tissue organs including brain, liver and spleen, but not for femur, mandible, and vertebrae, which is consistent with the published results^16^ (Supplementary Information, Figure S4a3 to f3). uDISCO method achieved excellent transparency for many organs, but was less effective for heavily colorized organs including liver and spleen^9^ (Supplementary Information, Fig. S4a4 to f4). The PEGASOS method achieved complete transparency for all above-mentioned organs (Supplementary Information, Figure S4a5 to f5).

Although PEGASOS method did not lead to shrinkage of bone samples we tested, we noticed that it caused shrinkage of the soft tissue organs. The shrinkage ratio varied between 30% (heart, liver) and 40% (brain, spleen) (Supplementary Information, Figure S5a). Analysis performed on brain slices indicates that the shrinkage is anistropic and does not cause detectable distortion of internal structures (Supplementary Information, Figure S5b).

### PEG component efficiently protects GFP and tdTomato fluorescence

**Fig. 5:**
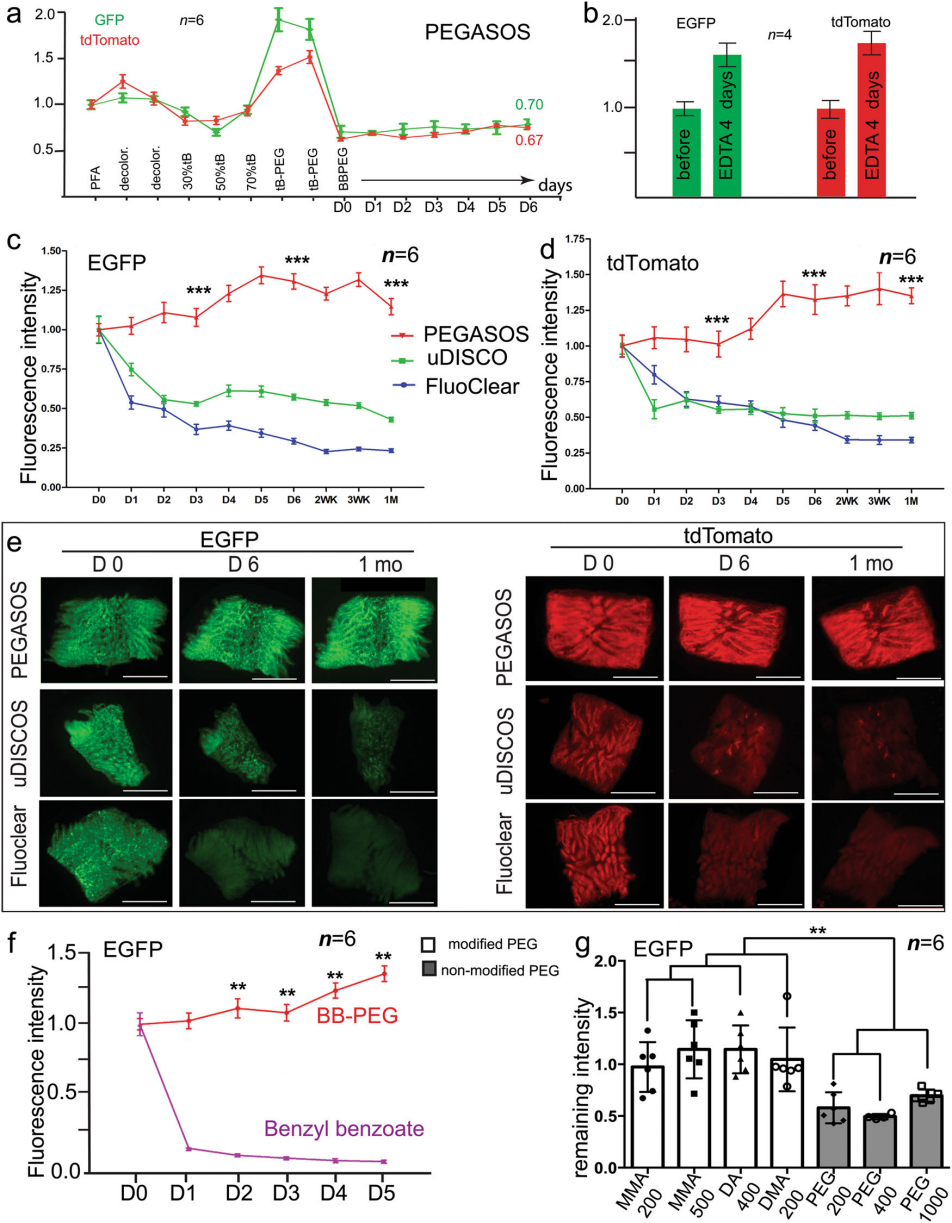
Modified PEG components within the BB-PEG medium preserves endogenous fluorescence for a long-term. Intestine samples were harvested from *CAG-EGFP* or *Tie2-Cre; Ai14* mice and cleared following indicated clearing methods. **a** GFP or tdTomato fluorescence intensity changes after each treatment step of PEGASOS. Twelve hours after fixation was recorded as the starting point. Each step lasted for 1 day. **b** Impact of 4 days of EDTA treatment on GFP or tdTomato fluorescence of fixed intestine samples. **c**, **d** GFP or tdTomato fluorescence intensity changes within different clearing media. We record 1 h after the samples being placed into the clearing medium as D0. **e** Representative images at various time points were acquired with the same exposure conditions. **f** Intestine samples were placed in complete BB-PEG medium or benzyl benzoate after the PEGASOS clearing for quantitative comparison. GFP fluorescence was preserved in BB-PEG medium, but rapidly quenched by benzyl benzoate. **g** To evaluate the impact of various forms of PEG on the fluorescence intensity, dehydrated intestine samples were placed in BB-PEG clearing medium of different formulations (benzyl benzoate (75% v/v) + various PEGs (25% v/v)). Fluorescence intensities were measured 1 week after clearing. All values are mean ± s.d. Statistical significance (**P < 0.01; *P < 0.05) was assessed by one-way ANOVA. Scale bars in **f**, 1 mm

Loss of endogenous fluorescence is a major concern for solvent-based tissue clearing medium. To evaluate the impact of clearing process on endogenous fluorescence, we performed quantitative assays using intestine samples harvested from either *CAG-EGFP* or *Tie2-Cre;Ai14* mice (P60). Intestine tissue was selected because large amount of tissue is available for parallel comparison and the thin tissue wall makes it more sensitive to chemical treatment. Samples were then processed following the PEGASOS immersion procedure for soft tissue organs. Each treatment step lasted for 1 day for evaluating the maximum effects. Fluorescence intensity after fixation was set as the original intensity. At the end of PEGASOS clearing process, both GFP and tdTomato fluorescence retained ~70% of the original intensity (Fig. 5a). Fluorescence intensity significantly increased during the delipidation and dehydration steps of PEGASOS, which was likely caused by increased auto-fluorescence (Fig. 5a). EDTA decalcification was commonly used for hard tissue research.^27^ Assays performed with intestine samples also indicated that 4 days of EDTA treatment did not compromise GFP or tdTomato fluorescence. Instead, the elution of PFA fixative by EDTA increased the sample fluorescence intensity (Fig. 5b).

Next, we evaluated and compared the preservation of endogenous fluorescence intensity in different clearing media. After the samples being placed in the BB-PEG medium, the endogenous fluorescence intensity increased in the first week and maintained at the same level even 1 month later. The final fluorescence intensity was 30%–50% higher than the beginning (Fig. 5c, d, e). In contrast, the GFP and tdTomato fluorescence intensities were reduced by around 50% 1 week after the treatment of the uDISCO or FluoClear clearing media (Fig. 5c, d, e).

The protection of fluorescence by the BB-PEG medium can be attributed to the presence of poly(ethylene glycol) (PEG) because pure benzyl benzoate immediately quenched GFP fluorescence (Fig. 5f). PEG is a large chemical family with many different forms.^28^ Among various PEGs we tested, PEGMMA and PEG diacrylate (PEGDA) provided the best protection for endogenous fluorescence. Less preservation effect was observed for unmodified PEG200, PEG400, and PEG1000 (Fig. 5g).

To evaluate the effects of PEGASOS clearing process on the auto-fluorescence of various types of tissues, brain slices (2 mm thickness) and masseter muscles (~1 mm thickness) were harvested from *Thy1-YFP-H* or *C57 Bl/6* mice of 2-months age. Calvarial bones were collected from *Tie2-Cre;Ai14* or *C57 Bl/6* mice of 2-months age. Samples were processed following the passive immersion procedure. Images were acquired either after fixation or after clearing with comparable exposure settings. Fluorescence was visualized on *Thy1-YFP-H* brain slice and *Tie2-Cre;Ai14* calvarial bone before and after clearing (Supplementary Information, Figure S6a, b, i, j). Neurons or vasculatures were visualized under higher magnification (Supplementary Information, Figure S6a’, b’, i’, j’). In contrast, no fluorescence was detected for brain and calvarial bone samples from *C57 Bl/6* mice after clearing (Supplementary Information, Figure S6k, k’, l, l’). Clearing of muscle tissues from *Thy1-YFP-H* mice provides better visualization of nerve fibers (Supplementary Information, Figure S6e, e’, f, f’). Comparing with brain and calvarial bone, muscle from a wild-type mouse presented stronger auto-fluorescence after clearing (Supplementary Information, Figure S6g, g’, h, h’).

### PEGASOS method is compatible with immunofluorescent staining

To evaluate if the PEGASOS method is compatible with immunohistochemical staining, we performed whole-mount immunofluorescent staining of various soft tissue organs and cleared them using the PEGASOS immersion method afterwards (Supplementary Information, Figure S7a). Anti-αSMA antibody staining displayed the arterial organization within the heart, kidney and spleen (Supplementary Information, Figure S7b–d). Laminin staining revealed the vasculature within a brain slice, as well as tubules and renal corpuscles within the kidney and intestinal villi (Supplementary Information, Figure S7f, g). Anti-GFAP antibody staining revealed the astrocytes within the brain (Supplementary Information, Figure S7h, h’). GS-IB4 staining revealed vasculatures within the mouse colon. (Supplementary Information, Figure S7i, i’). Anti-parvalbumin antibody staining revealed inhibitory neurons within the cerebellum (Supplementary Information, Figure S7j, j’). Anti-Collagen IV antibody staining revealed vasculatures within the mouse brain (Supplementary Information, Figure S7k, k’).

### Both PEGASOS recirculation and passive immersion procedures can be scaled up for clearing large animal models

To test if the PEGASOS can be scaled up for larger animal, we attempted to clear adult rats (12 weeks of age) with the recirculation procedure. Adult rats usually weigh over 300 grams and are tenfold heavier than adult mice. The entire clearing process took 1 month with duration of each step being doubled. The rat turned highly transparent after clearing process (Supplementary Information, Figure S8a, b). Body trunk was cleared with high transparency (Supplementary Information, Figure S8c). The rat mandible became nearly invisible (Supplementary Information, Figure S8d). Other organs including the femur, brain, heart, liver, spleen, and kidney were all efficiently cleared and showed complete transparency (Supplementary Information, Figure S8e-j).

We attempted to clear a fixed human brain sample (3 × 3 × 1 cm) with the PEGASOS passive immersion procedure. The human brain sample turned transparent after clearing (Supplementary Information Figure S9a, b). We performed laminin immunofluorescent staining for a human brain slice of 1 mm thickness and imaged it after clearing. Vascular networks within the human brain were clearly visualized (Supplementary Information, Figure S9c, c’).

A piece of dog tibia cortical bone (1 cm×3 mm×3 mm) was cleared following the passive immersion procedure for hard tissue. The bone piece turned nearly invisible after clearing (Supplementary Information, Figure S9d, e). The sample was imaged with a two-photon microscope. SHG signal could be detected even at 3 mm depth (Supplementary Information, Figure S9f-i**)**. Laminin staining was performed for a thin slice of bone sample (1 mm thickness) followed by clearing. Images acquired with a two-photon microscope revealed the Haversian Canals at the center of the osteons (Supplementary Information, Figure S9j).

### PEGASOS method enables whole-brain imaging and single axon tracing within the intact brain

**Fig. 6:**
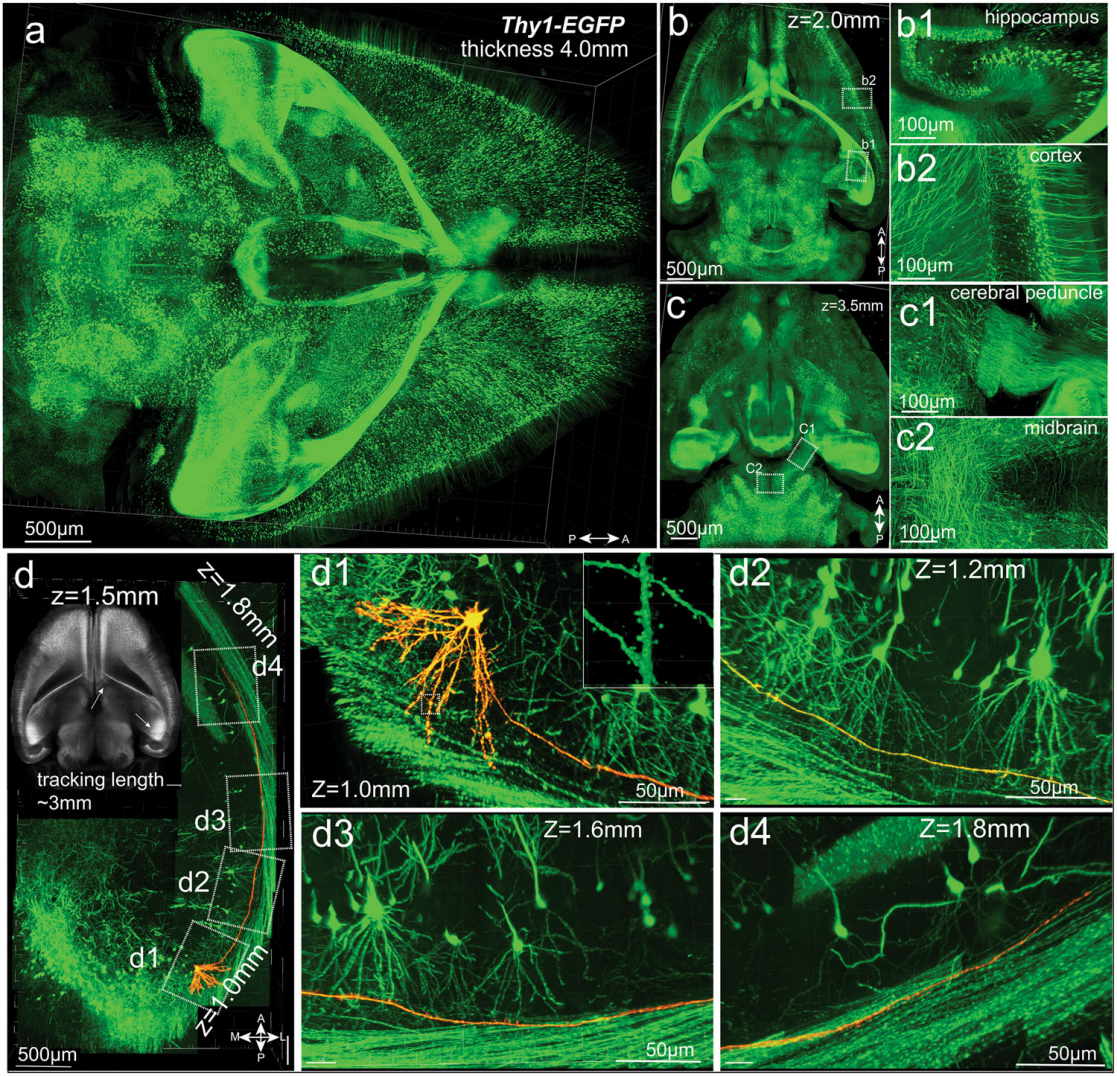
Whole-brain imaging and tracing of individual neurons and axons. Adult (60 days of age) *Thy1-EGFP* mouse brain was cleared following the PEGASOS passive immersion procedure. The dorsal-to-ventral thickness of the brain is around 4 mm after processing. **a** Whole-brain image acquired with a 10×/0.30 objective. Optical sections obtained at 2.0 mm and 3.5 mm are displayed in **b** and **c**. Boxed areas are enlarged to show hippocampus (**b1**), cortical neuron (**b2**), cerebral peduncle (**c1**) and midbrain (**c2**). Tracking individual axons in 3-D requires objective with higher NA to achieve high axial resolution. **d** Tiling images acquired with a 20×/0.95 objective on a confocal microscope show the tracking course of one neuron and its axon in 3-D. Inset at the corner was acquired with a 5× objective at the plane where the target neuron is located. Two arrows indicate the approximate beginning and ending positions of the tracking. Boxed areas are enlarged in panels **d1** to **d4**. Boxed area in **d1** is enlarged in the insert to show the dendritic spines. A, anterior; P, posterior; L, lateral; M, medial

Brains of adult *Thy1-EGFP* mice (60 days of age) were cleared following the passive immersion procedure for soft tissue organs. The dorsal-to-ventral thickness of the brain shrunk from ~6 mm to ~4 mm after processing. The brain has an obvious higher mechanical strength after clearing. We were able to reconstitute the entire brain with a 10×/0.30 objective on a confocal microscope by imaging from the dorsal-to-ventral direction. Neuron somas and axons were visualized throughout the entire brain including the cortex, hippocampus, cerebral peduncle and midbrain, indicating the sub-cellular resolution of the imaging throughout the entire brain (Fig. 6a, b, c, Supplementary Information, video 4).

We next explored the application of PEGASOS on tracing individual axons. Although the 10×/0.30 objective has a high lateral resolution of ~0.5 µm, its axial resolution is only ~10 µm,^29^ which is not sufficient for tracing the axons (~1 µm diameter) in 3-D. Therefore, we imaged intact *Thy1-EGFP* brain with a 20×/0.95 objective after clearing. We were able to identify individual neurons and trace one neuron for 3 mm starting from the hippocampus region to near the midline area along its axon within the corpus callosum. The tracing was interrupted due to the reason that the objective has a working distance limit of 1.95 mm (Leica 20×/0.95 WD1.95 mm) (Fig. 6d1–d4, Supplementary Information, video 5).

### High-speed acquisition of whole-brain vasculature image with the tiling light-sheet microscope

**Fig. 7:**
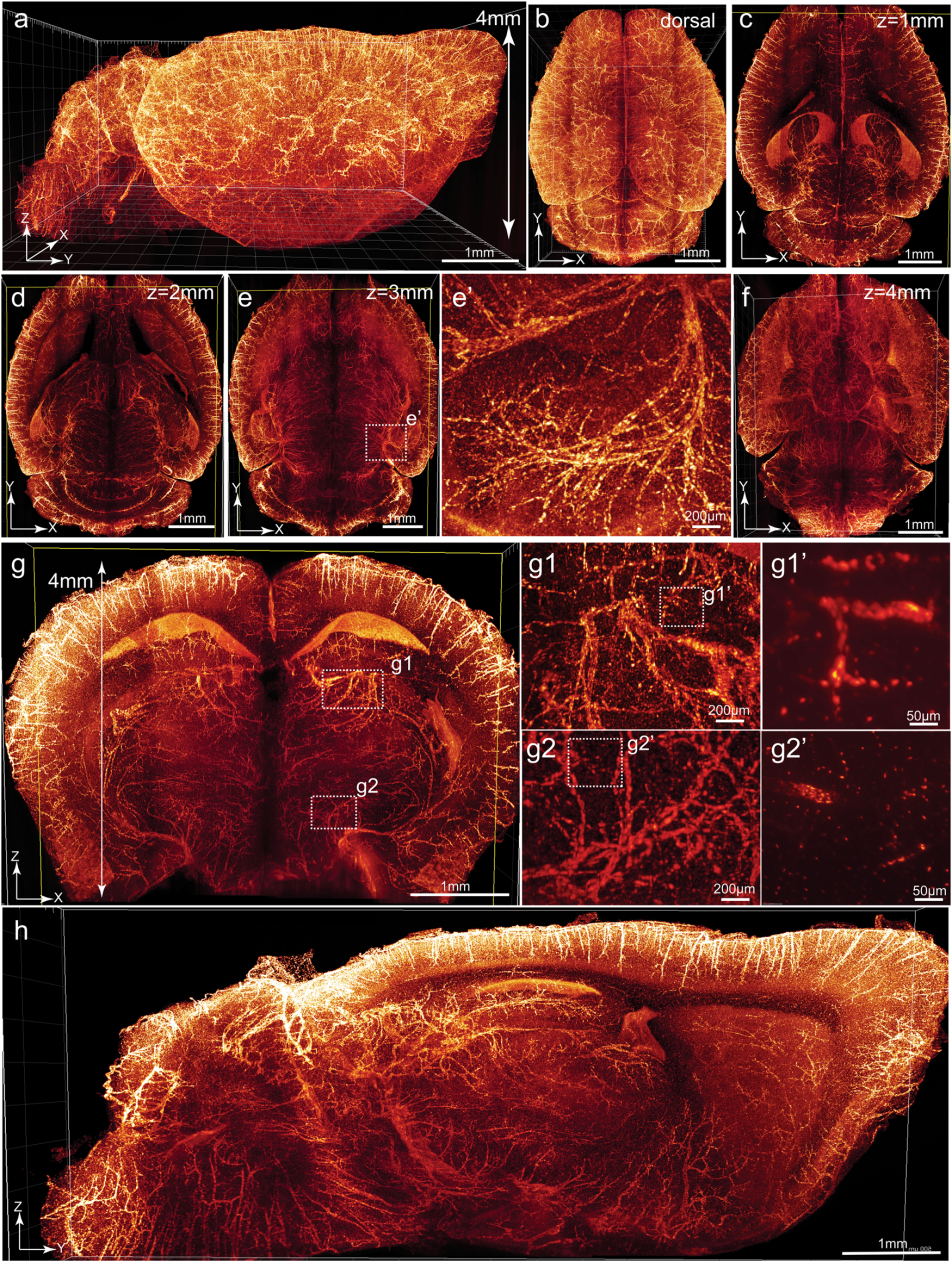
Imaging of the whole-brain vasculatures at sub-cellular resolution. Brains of adult *Tie2-Cre;tTA*^*flox*^*;tetO-H2BGFP* (*TTH*) mice were cleared and imaged with a tiling light-sheet microscope. Each dots represent nuclei of an endothelial cell. **a** A side view of the whole-brain image with dorsal-ventral thickness of ~4 mm. Dorsal view (**b**), optical sections acquired at 1 mm (**c**), 2 mm (**d**), 3 mm (**e**), and 4 mm (**f**) were displayed. Boxed region in **e** was enlarged in **e’**. **g** An X-Z optical slice acquired at the hippocampus position was displayed. Boxed areas in **g** were enlarged in **g1** and **g2**, respectively. Boxed regions in **g1** and **g2** were enlarged in **g1’** and **g2’**, respectively. **h** A sagittal Y-Z optical slice acquired at near the midline region

*Tie2-Cre;tTA*^*flox*^*;tetO-H2BGFP* mice (*TTH*) were generated for labeling the endothelial cells with nucleus-located H2BGFP. Size of the endothelium nuclei (~5 µm) provides reference for image resolution. Brains were harvested from *TTH* mice of P60 age and cleared following the PEGASOS passive immersion procedure. Cleared brains were imaged with a tiling light-sheet microscope.^30^ The entire brain was imaged within <5 h. Image stacks were acquired to display the brain from different angels (Fig. 7a, b, Supplementary Information, video 6). X-Y optical sections acquired at 1, 2, 3, and 4 mm showed high-resolution with individual nuclei being identifiable (Fig. 7c, d, e, e’, f). Endothelium nuclei could even be identified from coronal (Fig. 7g, g1, g2) and sagittal optical sections (Fig. 7h), suggesting the axial resolution of no less than 5 µm throughout the entire brain.

### PEGASOS method enables direct visualization of the DRG neurons within the vertebrae

Dorsal root ganglions (DRGs) relay peripheral sensory information into the CNS. In previous clearing studies, the spinal cord was dissected from the vertebrae for imaging due to the opaqueness of the bone.^9,20^ Connections between the DRG and spinal cord were not preserved in these studies.

**Fig. 8:**
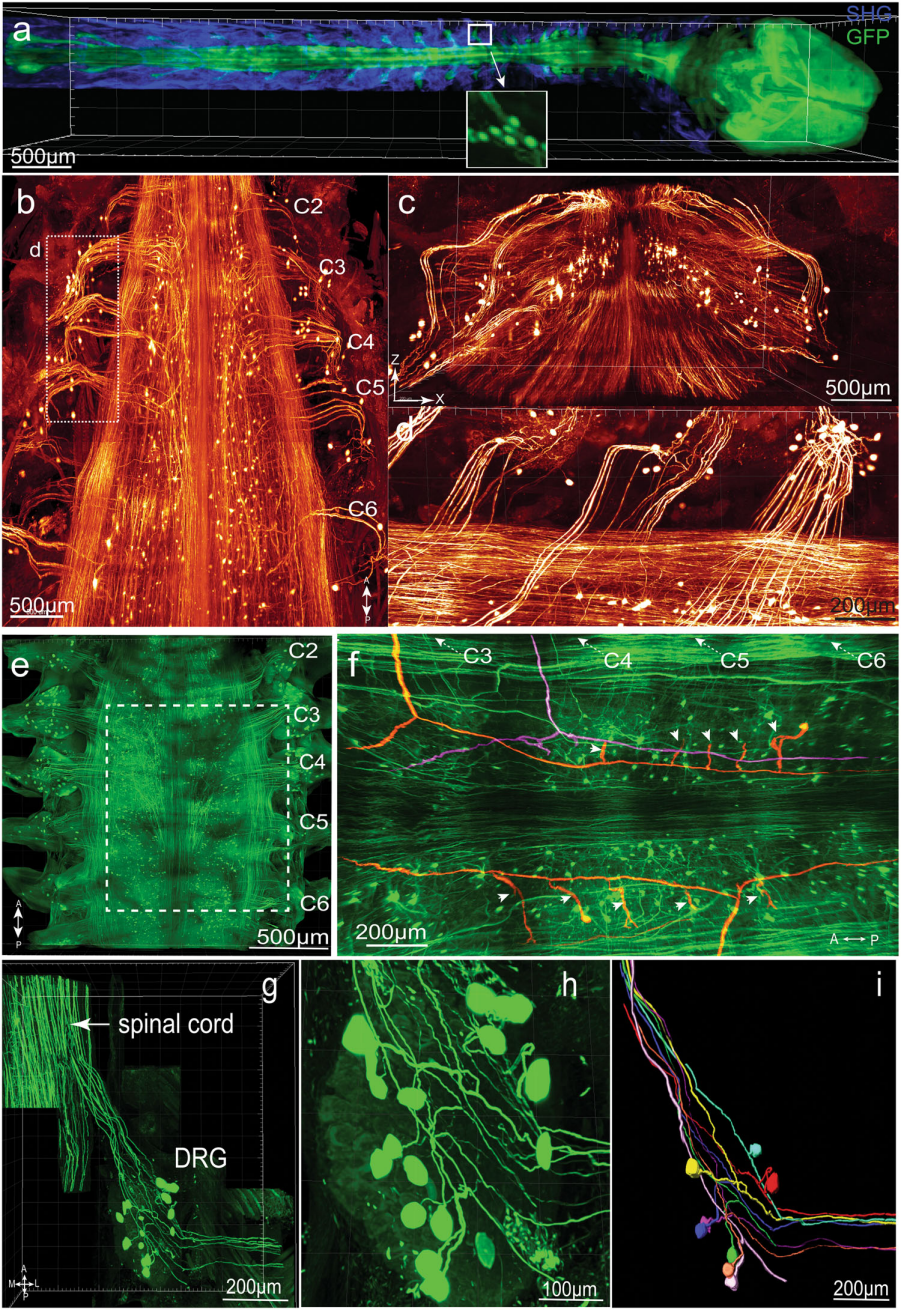
PEGASOS enables visualization of connections between CNS and PNS by imaging through the vertebrae. Adult *Thy1-EGFP* mice (2-months-old) were cleared following PEGASOS recirculation procedure and the vertebrae and brain were dissected for imaging. **a** The intact CNS together with DRGs were imaged with a 5×/0.16 objective on a two-photon microscope. Boxed area is enlarged in the insert to show individual DRG neurons. **b** A segment of cervical vertebrae (C2–C6) was imaged with a 1× 0.25NA objective on a tiling light-sheet microscope. X-Z optical slice was shown in **c** to display the cross section of the spinal cord. Boxed area in **b** was enlarged in **d** to display DRG neurons and their central axons**. e** A segment of the cervical vertebrae (C2–C6) was imaged with a 10×/0.45 objective on a confocal microscope to visualize cervical DRGs. **f** Optical sections were acquired at boxed region in **e**. Individual central axons were labeled. Each central axon (highlighted with color) gives rise to two daughter branches and then to multiple collateral branches (arrowheads). The entire tracing length is over 2 cm. **g–i** The C4 DRG was re-imaged with a 20×/0.95 objective to reveal connections between DRG neurons and the spinal cord. **h** Enlarged view shows individual DRG neurons within the C4 DRG. **i** Eight pseudo-unipolar neurons were individually identified and artificially labeled with different colors. SHG second harmonic generation signal. A, anterior; P, posterior; L, lateral; M, medial

We cleared adult *Thy1-EGFP* mice following the PEGASOS recirculation procedure. Due to the size limitation of the microscope stage, the vertebrae and brain were isolated for imaging. We were able to image the entire CNS together with DRGs through the vertebrae, thereby preserving these delicate connections (Fig. 8a). A segment of cervical vertebrae (C2–C7) was imaged with a 1×/0.25 objective on a tiling light-sheet microscope (Fig. 8b, Supplementary Information, video 7). Individual axons and neurons could be visualized on both Z-X and X-Y optical sections (Fig. 8c, d). The cervical vertebrae segment C2–C6 was imaged with a 10×/0.45 objective on a confocal microscope (Fig. 8e). We were able to trace individual central axons together with their daughter branches for over 2 cm within the spinal cord (Fig. 8f, Supplementary Information, video 8).

To achieve better resolution, we re-imaged C4 DRG with a 20×/0.95 objective. We were able to distinguish the origin and bifurcation of every axon within the DRG and artificially label each of the pseudo-unipolar neurons together with their axons with different colors (Fig. 8g, h, i, Supplementary Information, video 9).

### Visualization of neural and arterial networks within the marrow space of long bone

**Fig. 9:**
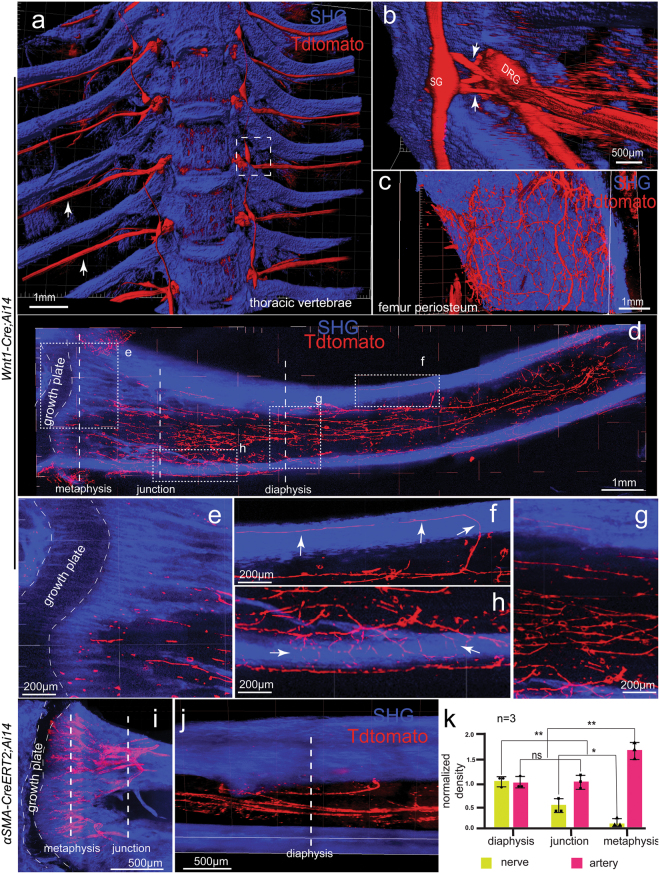
Nerves and arteries have distinct distribution patterns within the marrow space of long bone. **a** Thoracic cage from adult *Wnt1-Cre;Ai14* mouse (2-months-old) was cleared following the PEGASOS recirculation procedure and imaged with a two-photon microscope to display thoracic vertebrae, ribs, sympathetic trunks, and spinal nerves (arrows) exiting in the intervertebral foramina. **b** Dotted box in **a** is enlarged to display sympathetic ganglion (SG), DRG and the communication ramus (arrows). **c** Nerve bundles in the femur periosteum. **d** Maximum Z-projection of the tibia (~1.5 mm thickness) imaged with a 10× objective shows the innervation within the bone marrow. Dotted areas are enlarged in **e–h**. Arrows in **g** and **h** indicate nerve fibers penetrating the cortical bone. An intact tibia from *αSMA-Cre*^*ERT2*^*;Ai14* mice was cleared and imaged with a two-photon microscope to visualize the arterial organization within the bone marrow near the metaphysis region (**i**) and the mid-shaft diaphysis region (**j**). **k** Nerves and arteries densities at different areas within the marrow space were quantified and normalized. Sampling locations are indicated with dotted lines in **d, i**, and **j**. SHG, second harmonic generation signal. All values are mean ± s.d., Statistical significance (*P < 0.05; **P < 0.01) was assessed by one-way ANOVA

Nerves were known to be present within the bone marrow, but its distribution in 3-D has never been demonstrated. We used the *Wnt1-Cre;Ai14* mouse model for the labeling of the PNS. A thoracic segment was isolated from a *Wnt1-Cre;Ai14* mouse after clearing with the PEGASOS recirculation procedure. We imaged the sample with a 5×/0.16 objective. The SHG signal indicated the thoracic vertebrae and ribs. Sympathetic ganglia, DRG, spinal nerves, and communication rami in between were also visualized, suggesting efficient labeling of both sympathetic and sensory nerves (Fig. 9a, b). The intact tibia was cleared with the passive immersion procedure. Nerve fibers were visualized on the bone surface (Fig. 9c). We also visualized the neural network within the tibia bone marrow space (Fig. 9d, Supplementary Information, video 10). The major nerve bundles penetrate the cortical bone in the diaphysis region to enter the marrow space and branch out towards the growth plate (Fig. 9e–h). We used the *αSMA-Cre*^*ERT*^*;Ai14* mouse model to investigate the arterial organization of the tibia marrow space. Major arterial branches could be seen in the shaft region of the tibia and then branched out towards the growth plate, but no artery was seen penetrating it (Fig. 9i, j). Interestingly, quantification indicates that nerves are more enriched in the middle shaft region and are nearly absent from the trabecular bone, whereas the artery density is higher near the growth plate and lower in the diaphysis area (Fig. 9k).

### Investigation of mesenchymal stem cells (MSCs) response towards chemotherapy using tissue clearing-based 3-D imaging

Chemotherapy for leukemia patients was known to damage mesenchymal stem cells (MSCs) in vitro, but the extent to which MSCs are damaged by chemotherapy drugs in vivo was largely unknown.^31^ Cytarabine (Ara-C) is a chemotherapy medication for treating leukemia and non-Hodgkin’s lymphoma.^32^ Mouse incisor is an excellent model for studying MSCs in vivo. Its MSCs reside in the cervical loop region and give rise to active dividing transit amplifying cells (TA cells), which can be easily identified through EdU incorporation assay.^33^ 3-D imaging indicated that EdU + TA cells are more enriched at the cervical loop region of the incisor mesenchyme with a circular distribution pattern in 3-D (Supplementary Information, Figure S10a, b**)**. To investigate the impact of chemotherapy on MSCs, Ara-C was injected to adult mice for 7 consecutive days. 3-D quantification indicates that incisors of PBS-treated mice contain ~7000 TA cells in their mesenchyme. In contrast, TA cell number in incisors of AraC-treated mouse reduced to ~3000. The distribution patterns of TA cells in the two groups show no significant difference **(**Supplementary Information, Figure S10c, d, e**)**. These results suggest that AraC treatment inhibits the activation of incisor MSCs into TA cells.

## Discussion

PEGASOS achieved transparency for nearly all types of tissues including the teeth, long bone, spleen, liver, and heart. Pigment epithelium like retina remains to be the only tissue that cannot be cleared by the PEGASOS method. The decolorization treatment and high RI of the BB-PEG medium (RI of 1.54) are the main reasons for the high transparency of soft tissue organs including heart, kidney, spleen, and liver.^16^ The combination of Quadrol with ammonium provides better decolorization outcome. Decalcification treatment plays a critical role in clearing hard tissue organs including long bones and teeth. The successful clearing of the hard tissue provides a novel way for studying the hard tissue in 3-D in addition to traditional µCT. Tissue clearing and imaging method provides higher resolution than µCT and can be combined with multiple fluorescence labels.

Solvent-based tissue clearing techniques were known for superior transparency but poor fluorescence preservation. For the most recently published uDISCO method, the endogenous fluorescence was lost by over 50% 1 month after clearing.^9,17^ PEGASOS method achieved lossless preservation of endogenous GFP or tdTomato fluorescence. PEG component plays critical roles in the preservation. The amphiphilic PEGs may gather around GFP proteins and form an amenable interface to protect proteins from the denaturing effects of hydrophobic solvent molecules.^34–36^ This may explain the rise of fluorescence intensity in the first week after clearing. Addition of PEG gradually restores the fluorescent activity by renaturing the GFP and tdTomato proteins from previous delipidation and dehydration treatment. Additional protection might come from the modification groups on PEGs because unmodified PEGs showed weaker effects of protection. The double bonds within the modification groups may function as the reductant and react with the free radical groups in the solvent that quenched GFP protein.^37^ Moreover, the protective effect of the PEGASOS method may arise from the 3% Quadrol component. Adding 3% Quadrol to the tert-butanol solution and clearing medium maintains the pH over 9.5 and prevents the protonation of GFP proteins.^17^

DRGs are the relay ganglia between the CNS and PNS. Regeneration of the DRG central branches into the spinal cord is the most challenging issue after the spinal cord injury.^38^ Connection between DRGs and the spinal cord has never been demonstrated by any previous clearing methods.^9,25^ PEGASOS method renders the bone transparent and, therefore, we were able to directly image the spinal cord and DRG with single axon resolution without dissection. This capability will facilitate the spinal cord neural circuit analysis and regeneration studies.

Mapping neural circuits across the entire brain is a core mission of modern neuroscience^39^ and also a major driving force for developing various tissue clearing techniques. Different neurons within a single region may have heterogeneous projection patterns and carry diverse information.^40,41^ Tracing an individual axon in 3-D constitutes a major challenge not only for the tissue clearing technique, but also for the microscopy because it requires high lateral and axial resolution. Although we were able to image the whole brain with high lateral resolution using a 10×/0.30 objective, the insufficient axial resolution (~10 µm) made it very difficult to trace single axons in 3-D. We found 20× oil immersion objective with high NA (NA > 0.80) to be optimal for axon tracking, which has the lateral resolution of ~0.25 µm and axial resolution of ~3 µm. However, a full course tracing was still impeded by the limited working distance of the 20× immersion objective designed for high RI medium (1.95 mm for Leica 20×/0.95 and 0.57 mm for Zeiss 25×/0.8). Reduced SNR and increased optical aberration in the deep region are also important reasons for incomplete tracing.

The bone marrow is the primary location hosting hematopoietic stem cells (HSCs) and osteogenic mesenchymal stem cells (MSCs).^42^ Neural and periarterial niches critically regulate stem cell populations within the bone marrow.^33,43,44^ Although sparse nerve fibers within the bone marrow have been shown to be on tissue sections,^45^ their overall distribution pattern and densities have never been demonstrated due to the technical limitation on the bone marrow deep imaging. By imaging through the intact long bone, we were able to visualize the neural network within the bone marrow in 3-D. Although nerves were known to accompany arteries, their distribution densities within the bone marrow present different patterns. Nerves are more enriched in the shaft region, but are nearly absent from the trabecular bone area, where the osteogenesis resides. Arteries are more enriched at the trabecular bone region than at the shaft region. Our results indicate that the neural and periarterial niches do not overlap with each other and may have distinct roles on regulating stem cells. Further investigations will be needed to distinguish the different types of nerve fibers within the bone marrow because the *Wnt1-Cre* model labels both sympathetic and sensory nerves.^46^

Investigation of TA cell number reduction upon the administration of AraC, a commonly used chemotherapy drug, provides an example of the tissue clearing application on studying pathological conditions. The 3-D imaging based on PEGASOS clearing method enabled us to look inside the hard tissue to quantify the activation of mesenchymal stem cells. The 3-D imaging provides not only quantitative information, but also information about spatial pattern of cells, which is impossible for traditional approaches.

Soft tissue shrinkage remains to be the major drawback of the PEGASOS method. Although shrinkage does not lead to organization change inside of the organ, it can cause the detachment of brain from the calvarial bone. Shrinkage also significantly increases the auto-fluorescence of muscle tissue, which deteriorates the signal-to-noise ratio of the image. In addition, PEGASOS clearing method takes longer accomplishing time as compared with 3DISCO and uDISCO, due to the incorporation of decalcification and decolorization steps. Extra treatment steps might also make the method complicated to perform for regular labs. Despite of the progress of numerous tissue clearing techniques, signal aberration, and intensity reduction in the deep region still remain to be major challenges in whole organ 3-D imaging. Serial block face imaging combining tissue clearing and sectioning technique should be a promising direction in the field.

## Materials and methods

### Animals

Adult mice (6–8-weeks-age), both males and females, with genotypes including *C57BL/6* (JAX # 000664), *Thy1-YFP-H* (JAX 003782), *Thy1-eGFP-M* (JAX 007788), *Wnt1-Cre* (JAX022137), *αSMA-Cre*^*ERT2*^, *Tie2-Cre* (JAX 008863), *Ai14* (JAX 007908), and *CAG-EGFP* (JAX 003291) were used in the experiments. Sprague Dawley rats (300 g) (Charles River 400) of both males and females were used for experiments. Dog tibia bone samples were kindly provided by Dr. Jian Q. Feng of the Texas A&M University. Human brain tissue samples were kindly provided by Dr. Woo-Ping Ge of the UT Southwestern Medical Center. All animal experiments were approved by the Institutional Animal Care and Use Committee of UT Southwestern Medical Center and the Texas A&M University, and were in accordance with guidelines from the NIH/NIDCR.

### Preparation of PEGASOS solutions

#### Decalcification solution

Decalcification solution was prepared by mixing 20% w/v ethylenediaminetetraacetic acid (EDTA) (Sigma-Aldrich 324503) with H_2_O. Sodium hydroxide (Sigma-Aldrich S8045) was added to adjust the pH to 7.0.

#### Decolorization solutions

Quadrol (Sigma-Aldrich 122262) was diluted with H_2_O to a final concentration of 25% v/v. Ammonium (Sigma-Aldrich 105432) was diluted with H_2_O to a final concentration of 5% v/v.

#### Gradient tB delipidation solution

Pure *tert*-Butanol (tB) (Sigma-Aldrich 360538) was diluted with distilled H_2_O to prepare gradient delipidation solutions: 30% v/v, 50% v/v and 70% v/v. Quadrol (Sigma-Aldrich 122262) was then added with 3% w/v final concentration to adjust the pH to above 9.5.

#### tB-PEG dehydration solution

Dehydrating solution was composed of 70% v/v *tert*-Butanol, 27% v/v PEG methacrylate M_n_ 500 (PEGMMA500) (Sigma-Aldrich 409529) and 3% w/v Quadrol (Sigma-Aldrich 122262).

#### BB-PEG clearing medium (refractive index R.I. 1.543)

BB-PEG was prepared from mixing 75% v/v benzyl benzoate (BB) (Sigma-Aldrich B6630) and 25% v/v PEGMMA500 (Sigma-Aldrich 409529) supplemented with 3% w/v Quadrol (Sigma-Aldrich 122262) together. The fresh medium was a colorless liquid with low viscosity and turned slightly yellow in a week. Other forms of PEGs including PEGMMA200 (Polysciences 16664), PEGDA400 (Polysciences, 01871), PEGDMA (Polysciences, 00096), PEG200 (Sigma-Aldrich 81150), PEG400 (Sigma-Aldrich 807485), and PEG1000 (Sigma-Aldrich 81190) were also used for comparing their fluorescence preservation capabilities.

### Perfusion and tissue preparation

Before transcardiac perfusion, mice were anesthetized with an intraperitoneal injection of a combination of xylazine and ketamine anesthetics (Xylazine 10-12.5 mg/kg; Ketamine, 80–100 mg/kg body weight). For mice, 50–100 ml ice-cold heparin PBS (10 U/ml heparin sodium in 0.01 M PBS) was injected transcardially to wash out the blood. In all, 50 ml 4% PFA (4% paraformaldehyde in 0.01 M PBS, pH 7.4) was then infused transcardially for fixation. For rats, a circulation pump (VWR 23609-170) was applied to supply sufficient perfusion pressure and speed, and 500–1000 ml fresh heparin PBS and 500 ml 4% PFA were circulated to perfuse.

For the whole-body tissue clearing procedure, the skin, eyeballs, and tongue were removed. The contents of the stomach and gut were flushed out with PBS. For immersion of individual organs, the organs under study were dissected and immersed in 4% PFA at room temperature for 24 h before proceeding to tissue clearing.

### Whole-body clearing with the PEGASOS recirculation procedure

Immediately following standard transcardiac perfusion with heparin PBS (10 U/ml heparin sodium in 0.1 M PBS), the mice were perfused with 4% PFA (in PBS, pH 7.4) and fixed at room temperature for 12 h. The mice were then transferred into a perfusion chamber at 37 °C for recirculation of all reagents sequentially driven by a peristaltic pump (VWR 23609-170). The mice were perfused with 20% EDTA (pH 7.0) for 4 days for decalcification and then pure water (pH 7.0) for 2 h for washing off the resulting salt. Twenty-five percent after that, the mice were perfused with After that, the mice were perfused with 25 % Quadrol decolorization solution for 1 day for decolorization. In all, 30% v/v, 50% v/v and 70% v/v tB delipidation solutions were sequentially infused to mice for delipidation for 1 day per concentration. The mice were then dehydrated with the tB-PEG medium for 2 days with daily medium change. The mice were finally perfused with the BB-PEG clearing medium until the tissue turned transparent, which usually took at least 24 h. A typical recirculation clearing process took 2 weeks. Samples can be preserved in the BB-PEG clearing medium at room temperature.

For clearing adult rats ( > 300 g) with the recirculation procedure, the duration for each step was doubled and the entire clearing process took 1 month.

### PEGASOS passive immersion procedure

For clearing hard tissue samples, 4% PFA fixation was performed at room temperature for 12 h and then samples were immersed in 20% EDTA (pH 7.0) at 37 °C in a shaker for 4 days. Samples were then washed with H_2_O for at least 30 min to elute excessive EDTA. Following that, samples were decolorized with the Quadrol decolorization solution for 2 days and 5% ammonium solution at 37 °C in a shaker. Samples were placed in gradient tB delipidation solutions for 1–2 days and then tB-PEG for 2 days for dehydration. Samples were then immersed in the BB-PEG medium at 37 °C for at least 1 day for clearing.

For soft tissue or tissue slices, decalcification procedure was skipped. After fixation with 4% PFA solution for 24 h, samples were treated with Quadrol decolorization solution for 2 days at 37 °C. Samples were then immersed in gradient delipidation solutions at 37 °C shaker for 1 to 2 days, followed by dehydration solution treatment for 1 to 2 days and BB-PEG clearing medium treatment for at least 1 day until reaching transparency. Samples were then preserved in the clearing medium at room temperature.

The time schedule for clearing different types of tissue with immersion method is summarized (Supplementary Information, Table S1). In addition, a detailed step-by-step working protocol for passive immersion method is also provided (Supplementary Information, Data S1).

### Measurement of the organ size change after treatment

Dissected organ samples before and after treatment were imaged with a stereomicroscope (Olympus SZX16) under the same magnification. Sample area was outlined and quantified using Image J (NIH). The area after treatment was divided by the area before treatment for normalization.

### Vasculature labeling with the isolectin GS-IB_4_ dye

Adult mice of 6–8 weeks of age were placed in a restrainer after anesthesia. The tail was warmed with a heat lamp for about 1 min and then wiped with 70% ethanol around the injection site. A 30 G needle was inserted with the bevel up, going 5–15 degrees into the vein. Two hundred microliters of 500 µg/ml AlexFluo568 conjugated GS-IB_4_ (Thermofisher,121412) was injected. Mice were sacrificed 10 min later with CO_2_ for sample collection.

### Fluorescence intensity quantification

Intestine slices (2 X2 X0.5 mm) were harvested from adult *CAG-EGFP* or *Tie2-Cre;Ai14* mice as the test samples. Fluorescent images were captured with a stereo fluorescence microscope (Zeiss AxioZoom.V16) with the same imaging parameters for all testing groups. Fluorescence intensity values were measured using Image J (NIH).^47^ When we evaluate the effects of treatment steps on fluorescence intensities, the signal intensity of sample after PFA fixation was set as the initial value. The “integrated intensity” values were measured. When we compare the fluorescence preservation in different clearing media, the initial time point, D0, was set at 1 h after immersing samples into the clearing medium. Afterwards, measurements for the “mean gray value” were made at indicated time points. The D0 fluorescence value was normalized as 1.00 and the relative fluorescence intensity was shown as the ratio of fluorescence intensity at other time points to fluorescence intensity at D0. When we evaluate the fluorescence preservation properties of different PEGs, the fluorescence intensity ratio (mean gray value) of D7 to D0 was used to evaluate the remaining fluorescence intensity.

### Neural and arterial density calculation

Calculation of neural and arterial density was performed on the 3-D reconstituted tibia imaged with a 20× objective. Adult *αSMA-Cre*^*ERT*^*;Ai14* mouse model (P60) was used for labeling arteries. Adult *Wnt1-Cre* mouse model (P60) was used for labeling nerves. Measurement was performed in three areas including diaphysis, metaphysis, and the junction area between them. For each area, at least three samples were used for quantification. Numbers of vessels or nerves were counted in unit volume. The density value is given as number of nerves or arteries/Unit volume (µm^3^). The density value in diaphysis was normalized as 1.0. The density values in other regions were divided by the density value in diaphysis for normalization. Statistical analysis was performed using one-way ANOVA and Tukey’s multiple comparison test.

### Microscopy and image analysis

Whole-body and whole-organ fluorescent images were acquired with Zeiss AxioZoom V16 Stereomicroscopy, Zeiss LSM 780 two-photon microscopy (Visible laser lines: 405, 458, 488, 514, 561, 594, 633 nm; Coherent Vision II laser for two-photon imaging; Spectral detection with ultrasensitive GaAsP detector), Zeiss LSM 880 two-photon microscopy (Visible laser lines: 405, 458, 488, 514, 561, 594, and 633 nm for conventional confocal applications; Coherent Vision II laser for two-photon imaging; Dual channel, non-descanned detector for deep imaging and second harmonic generation (SHG) imaging), Leica TCS SP8 confocal microscopy (Laser lines: 405, 458, 488, 514, 568, 594, and 633 nm; Leica HyD^TM^ photon detector). Leica TCS SP5 confocal microscopy (Laser lines: 405, 458, 488, 514, 568, 594 and 633  nm; PMT detector).

A prototype tiling light-sheet selective plane illumination microscope (TLS-SPIM), designed and constructed by Dr. Liang Gao in the 3I Inc. (Intelligent Imaging Innovations, Inc., Denver, CO), was used to image the cleared *TTH* brain and *Thy1-EGFP* vertebrae samples.^30^ A tiling light-sheet tiled at multiple positions within the field of view was used to illuminate the sample, and the sample was scanned with a 1×/0.25NA objective axially at a ~2 µm step size to image the selected region of interests in 3-D. The microscope and objective parameters for acquiring figures are displayed in Supplementary Information, Table S2.

Imaging processing and 3-D rendering was performed with a Dell Precision T7600 workstation with dual Xeon 2670 processor, 128 GB RAM and AMD Radeon 480 graphic card.

All raw image data were collected in a lossless 16-bit TIFF format. Blind deconvolution processing was performed using Autoquant X3 (Media Cybernetics). Tiling of multiple image stacks were performed using Image J (NIH).^47^ 3-D reconstruction images and movies were generated using Imaris 9.0 (Bitplane). Stack images were generated using the “volume rendering” function. Optical slices were obtained using the “orthoslicer” function. 3-D pictures were generated using the “snapshot” function. Movies were generated using “animation” function.

### Whole-mount immunohistochemical staining

In all, 1.5 mm thick sections of various organs were used for whole-mount immunohistochemical staining. After fixation with 4% PFA for 24 h, samples were decolorized with 25% Quadrol for 1 day. Next, samples were washed with the PBS solution for 30 min. Samples were then immersed in the blocking solution composed of 10% dimethyl sulfoxide (Sigma-Aldrich 276855), 0.5% IgePal630 (Sigma-Aldrich 18896) and 1× casein buffer (Vector, SP-5020) in 1 ml 0.01 M PBS overnight at room temperature. After blocking, samples were stained with the primary antibody diluted with the blocking solution for 72 h at 4 °C on a shaker. Tissues were then washed with PBS at room temperature for 1 day. After that, samples were stained with the secondary antibodies diluted with the blocking solution for another 3 days at 4 °C on a shaker. PBS washing was performed for the samples for 6 h. Samples were then moved to the delipidation and dehydration solutions following the passive immersion procedure. After final clearing with the BB-PEG medium, samples were maintained in the clearing solution for imaging.

Antibodies used for whole-mount staining included anti-αSMA FITC antibody produced in mouse (dilution 1:500, Sigma-Aldrich F3777), rabbit anti-parvalbumin antibody (dilution 1:1000, Abcam ab11427), rabbit anti-GFAP antibody (1:100, Abcam ab7260), rabbit anti-Collagen IV antibody (1:100, Abcam ab6586), rabbit anti-Laminin antibody (dilution 1:50, Sigma-Aldrich L9393), goat anti-mouse IgG Alexa Fluor 488 and goat anti-rabbit IgG Alexa Fluor 488 (dilution 1:200, ThermoFisher A-11029 and A11034).

### AraC injection and EdU incorporation assay

Adult C57BL/6 mice littermates (7 weeks of age) were used for cytosine arabinoside (AraC) treatment experiment. AraC (Sigma-Aldrich, C1768) was injected intraperitoneally to mice in the treatment group mice once per day for 7 days at a dosage of 0.5 µg/10 g of mouse body weight. Edu (ThermoFisher Sci. E104152) was injected 2 h before sacrificing mice. Mandibles were collected and processed following the PEGASOS passive immersion procedure for the hard tissue. EdU whole-mount staining was performed after the decolorization step. Samples were immersed in the EdU labeling cocktail (ThermoFisher Sci. C10339) for 2 days under constant shaking and then washed with PBS for 2 h. Samples were then placed in the delipidation medium for continuing the clearing procedure until transparency was achieved.

All cleared mandibles were imaged with a 25×/0.8NA objective on the Zeiss LSM 880 two-photon microscopy. EdU + cell numbers within the incisor were quantified using the “surface function” in Imaris 9.0 (Bitplane).

### Quantification and statistical analysis

*N* numbers are reported in the figures and corresponding legends. Data are presented as mean ± standard deviation using Student’s *t*-tests or one-way ANOVA. Statistical analysis was performed in Microsoft Excel and GraphPad Prism.

## Electronic supplementary material

Supplementary information, Video S1

Supplementary information, Video S2

Supplementary information, Video S3

Supplementary information, Video S4

Supplementary information, Video S5

Supplementary information, Video S6

Supplementary information, Video S7

Supplementary information, Video S8

Supplementary information, Video S9

Supplementary information, Video S10

Supplementary information, Figure S2

Supplementary information, Figure S3

Supplementary information, Figure S4

Supplementary information, Figure S5

Supplementary information, Figure S6

Supplementary information, Figure S7

Supplementary information, Figure S8

Supplementary information, Figure S9

Supplementary information, Figure S10

Supplementary information, Table S1

Supplementary information, Data S1

Supplementary information, Figure S1

Supplementary video legend
