# Supplementary material for: Tissue clearing of both hard and soft tissue organs with the PEGASOS method
Source: Cell Res. 2018 May 29;28(8):803–18. doi: 10.1038/s41422-018-0049-z (PMC6082844; doi:10.1038/s41422-018-0049-z)
Supplement: Supplementary file 11 — Supplementary information, Figure S2 [file 41422_2018_49_MOESM11_ESM.pdf]

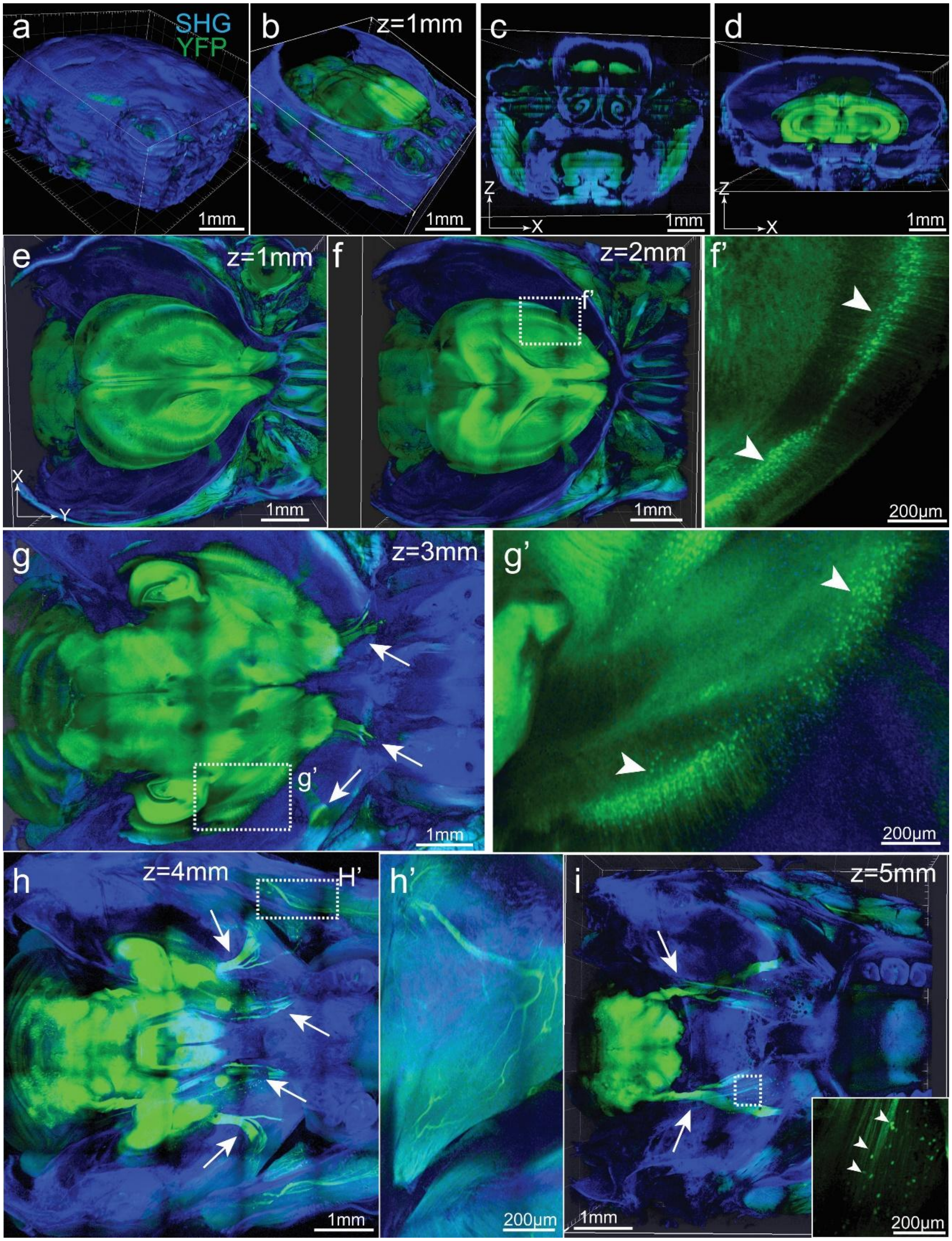

SHG
YFP
a
b
z=1mm
c
d
1mm
e
z=1mm
f
z=2mm
f'
200µm
g
z=3mm
g'
h
z=4mm
H'
h'
i
z=5mm

Figure S2. Whole skull imaging at single cell resolution. *Thy1-YFP-H* mice of P60 age were cleared following the full body recirculation procedure. The skull containing the brain was isolated and imaged with a 5X objective on a two-photon microscope. **(a)**. The entire image stack was displayed from side. **(b).** The side view of the image stack with optical section at Z=1mm to display the brain within the skull. Two coronal X-Z optical sections were acquired at nasal cavity **(c)** and brain region **(d)**. X-Y optical sections at Z=1mm **(e),** Z=2mm **(f),** Z=3mm **(g),** Z=4mm **(h)** and Z=5mm **(i)** were acquired. Boxed region is **(f)** was enlarged in **(f')** to show individual neurons within the cortex (arrowheads). Boxed region in **(g)** was enlarged in (**g'**) to show individual cortical neurons (arrowheads). Arrows in **(g, h** and **i)** indicate cranial nerves. Boxed region in **(h)** was enlarged to show nerves within the facial muscles. Boxed region in **(i)** was enlarged to show individual neurons within the trigeminal ganglion (arrowheads). SHG, Second harmonic generation signal.
