# Supplementary material for: Tissue clearing of both hard and soft tissue organs with the PEGASOS method
Source: Cell Res. 2018 May 29;28(8):803–18. doi: 10.1038/s41422-018-0049-z (PMC6082844; doi:10.1038/s41422-018-0049-z)
Supplement: Supplementary file 12 — Supplementary information, Figure S3 [file 41422_2018_49_MOESM12_ESM.pdf]

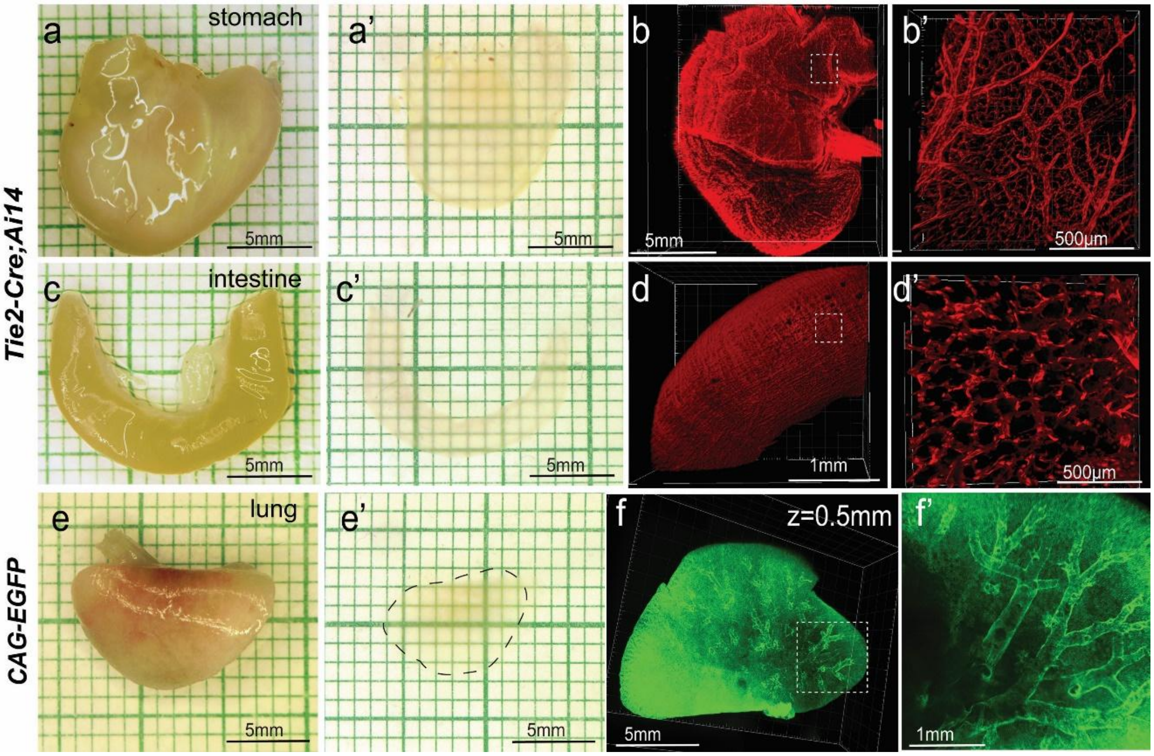
Tie2-Cre;Ai14
CAG-EGFP
a
stomach
5mm
a’
5mm
b
5mm
b’
500µm
c
intestine
5mm
c’
5mm
d
1mm
d’
500µm
e
lung
5mm
e’
5mm
f
z=0.5mm
5mm
f’
1mm

Figure S3. PEGASOS passive immersion procedure enables soft tissue organs clearing and imaging. Samples were harvested from adult mice (60 days age) of *Tie2-Cre;Ai14* or *CAG-EGFP* and processed following the passive immersion procedure. (a). Intact stomach of a *Tie2-Cre;Ai14* mouse was imaged before (**a**) and after clearing (**a'**). **(b)**. The cleared stomach was imaged with a confocal microscope. Boxed area is enlarged in **(b')** to show the vascular organization. **(c)**. A segment of intestine was cleared to complete transparency **(c')** and then imaged with a confocal microscope **(d)**. Boxed area is enlarged in **(d')** to display the vascular network. **(e).** An intact lung from a *CAG-EGFP* mouse was cleared **(e')** and imaged **(f)**. Boxed region is enlarged to display the branchial organization **(f')**.
