# Supplementary material for: Tissue clearing of both hard and soft tissue organs with the PEGASOS method
Source: Cell Res. 2018 May 29;28(8):803–18. doi: 10.1038/s41422-018-0049-z (PMC6082844; doi:10.1038/s41422-018-0049-z)
Supplement: Supplementary file 13 — Supplementary information, Figure S4 [file 41422_2018_49_MOESM13_ESM.pdf]

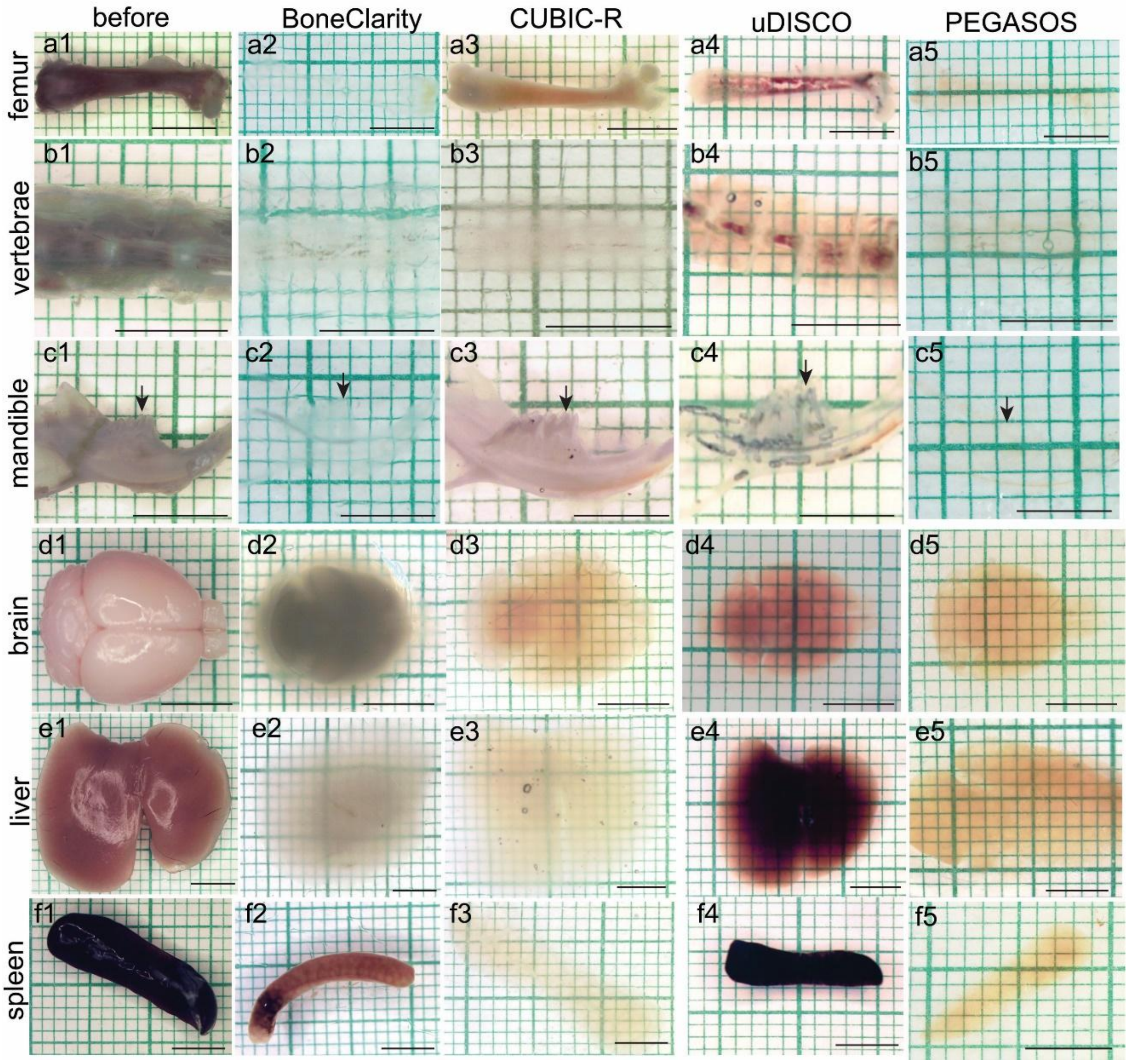
before
BoneClarity
CUBIC-R
uDISCO
PEGASOS
femur
vertebrae
mandible
brain
liver
spleen
a1
a2
a3
a4
a5
b1
b2
b3
b4
b5
c1
c2
c3
c4
c5
d1
d2
d3
d4
d5
e1
e2
e3
e4
e5
f1
f2
f3
f4
f5

**Figure S4. Comparison of clearing performance between the PEGASOS and other clearing methods**. Hard and soft tissue samples were harvested from adult C57BL/6 mice (P60) and processed following BoneCLARITY, CUBIC-R, uDISCO or PEGASOS passive immersion method[9,15,16]. Representative images were taken. Arrows in c1 to c5 indicate the mandibular first molar. Scale bars, 5mm.
