# Supplementary material for: Tissue clearing of both hard and soft tissue organs with the PEGASOS method
Source: Cell Res. 2018 May 29;28(8):803–18. doi: 10.1038/s41422-018-0049-z (PMC6082844; doi:10.1038/s41422-018-0049-z)
Supplement: Supplementary file 14 — Supplementary information, Figure S5 [file 41422_2018_49_MOESM14_ESM.pdf]

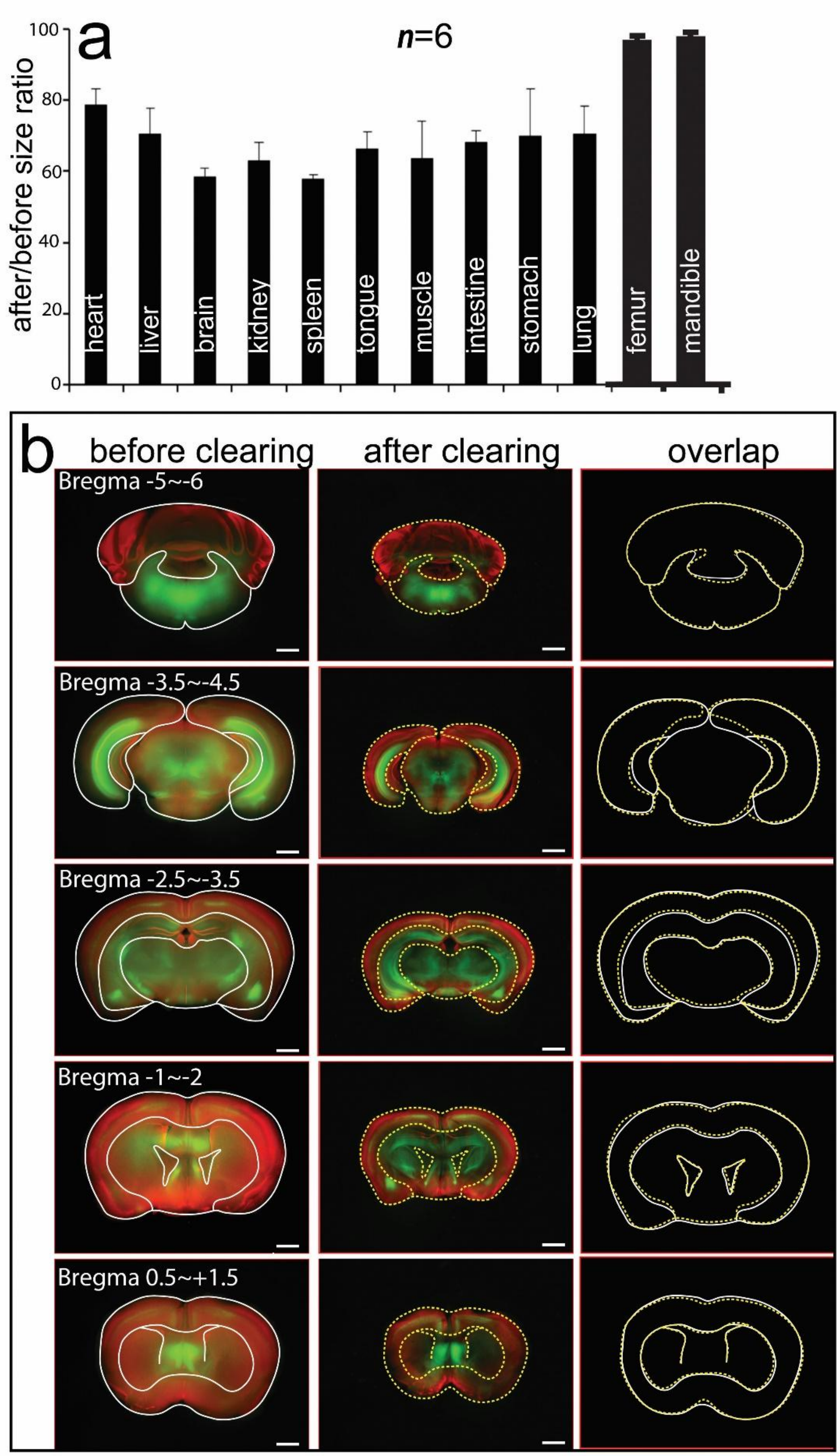
a
n=6
after/before size ratio
100
80
60
40
20
0
heart
liver
brain
kidney
spleen
tongue
muscle
intestine
stomach
lung
femur
mandible
b
before clearing
after clearing
overlap
Bregma -5~-6
Bregma -3.5~-4.5
Bregma -2.5~-3.5
Bregma -1~-2
Bregma 0.5~+1.5

Figure S5. Size change of organs after clearing. (a). Size change of various organs after clearing with PEGASOS passive immersion procedure. n=6. **(b)**. Brain Shrinkage is anistrophic. Coronal brain slices of 2mm thickness were harvested from *Thy1-EGFP* mice (60 days of age) at different levels and stained with propidium iodide. Sections were then imaged with a fluorescence stereomicroscope before and after PEGASOS clearing. The borders of major structures before and after clearing are highlighted in solid or broken lines respectively. The after-clearing border lines are ratio-metricly enlarged to compare with the before-clearing borders and they showed overlap. Scale bars, 1mm.
