# Supplementary material for: Tissue clearing of both hard and soft tissue organs with the PEGASOS method
Source: Cell Res. 2018 May 29;28(8):803–18. doi: 10.1038/s41422-018-0049-z (PMC6082844; doi:10.1038/s41422-018-0049-z)
Supplement: Supplementary file 15 — Supplementary information, Figure S6 [file 41422_2018_49_MOESM15_ESM.pdf]

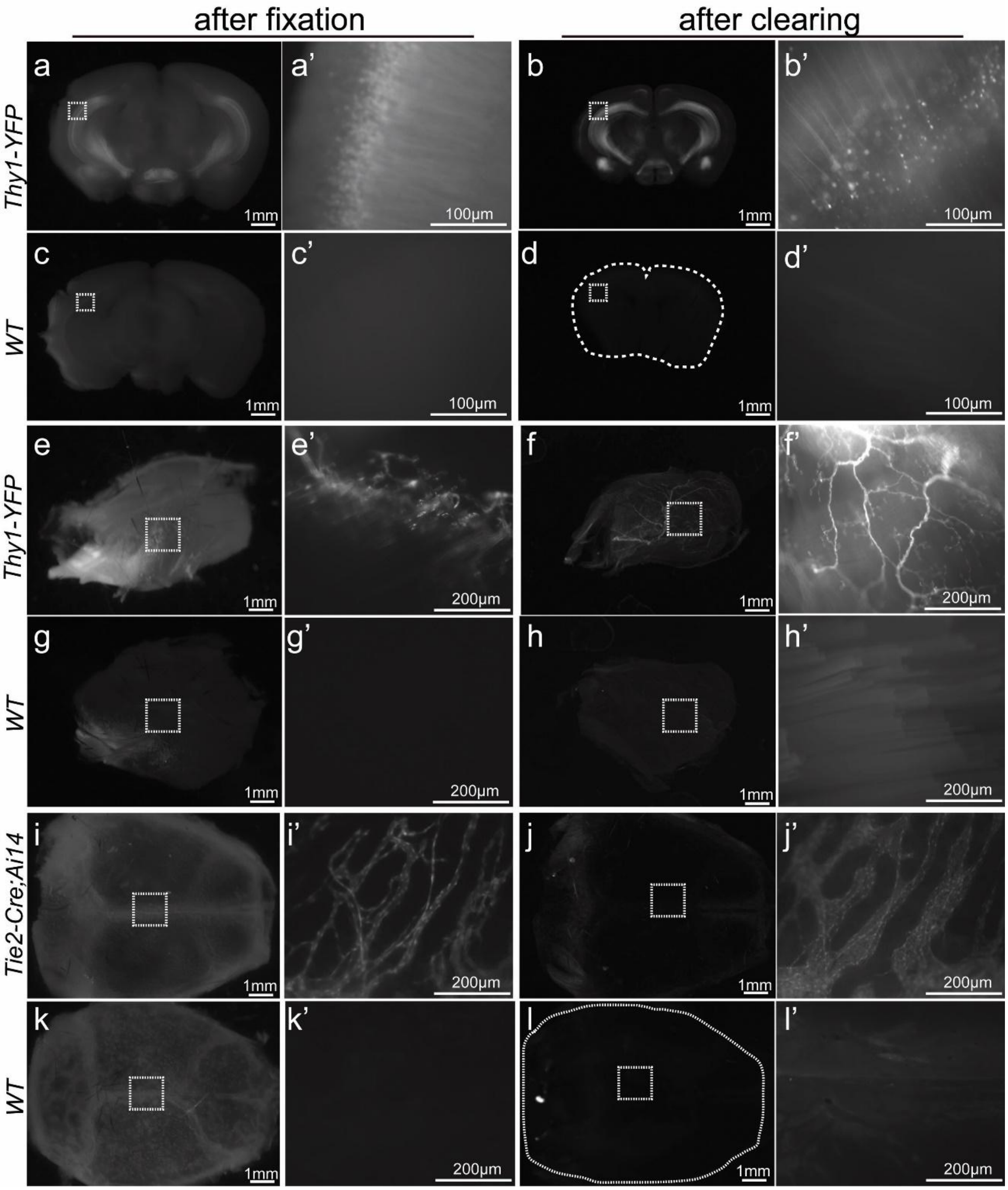
after fixation
after clearing
Thy1-YFP
WT
Thy1-YFP
WT
Tie2-Cre;Ai14
WT
a
a'
b
b'
c
c'
d
d'
e
e'
f
f'
g
g'
h
h'
i
i'
j
j'
k
k'
l
l'
1mm
100μm
200μm

Figure S6. Effects of PEGASOS clearing on the autofluorescence intensities of various types of tissue. (**a-d**). Brain slices (2mm thickness) were harvested from *Thy1-YFP-H* mice or *C57 Bl/6* mice (2 months age). (**e-h**). Masseter muscles (~1mm thickness) were dissected from *Thy1-YFP-H* mice or *C57 Bl/6* mice (2 months age). (**i-l**). Calvarial bones were harvested from *Tie2-Cre;Ai14* or *C57 Bl/6* mice (2 months age). Tissues were processed following the passive immersion procedure. Images were acquired with a stereo fluorescent microscope after fixation or after clearing. Comparable imaging parameters were used for all samples. (**a'-l'**). Boxed areas in the left panels were re-imaged at higher magnification. Dotted lines outline the nearly invisible brain slice (**d**) or calvarial bone (**l)**.
