# Supplementary material for: Tissue clearing of both hard and soft tissue organs with the PEGASOS method
Source: Cell Res. 2018 May 29;28(8):803–18. doi: 10.1038/s41422-018-0049-z (PMC6082844; doi:10.1038/s41422-018-0049-z)
Supplement: Supplementary file 16 — Supplementary information, Figure S7 [file 41422_2018_49_MOESM16_ESM.pdf]

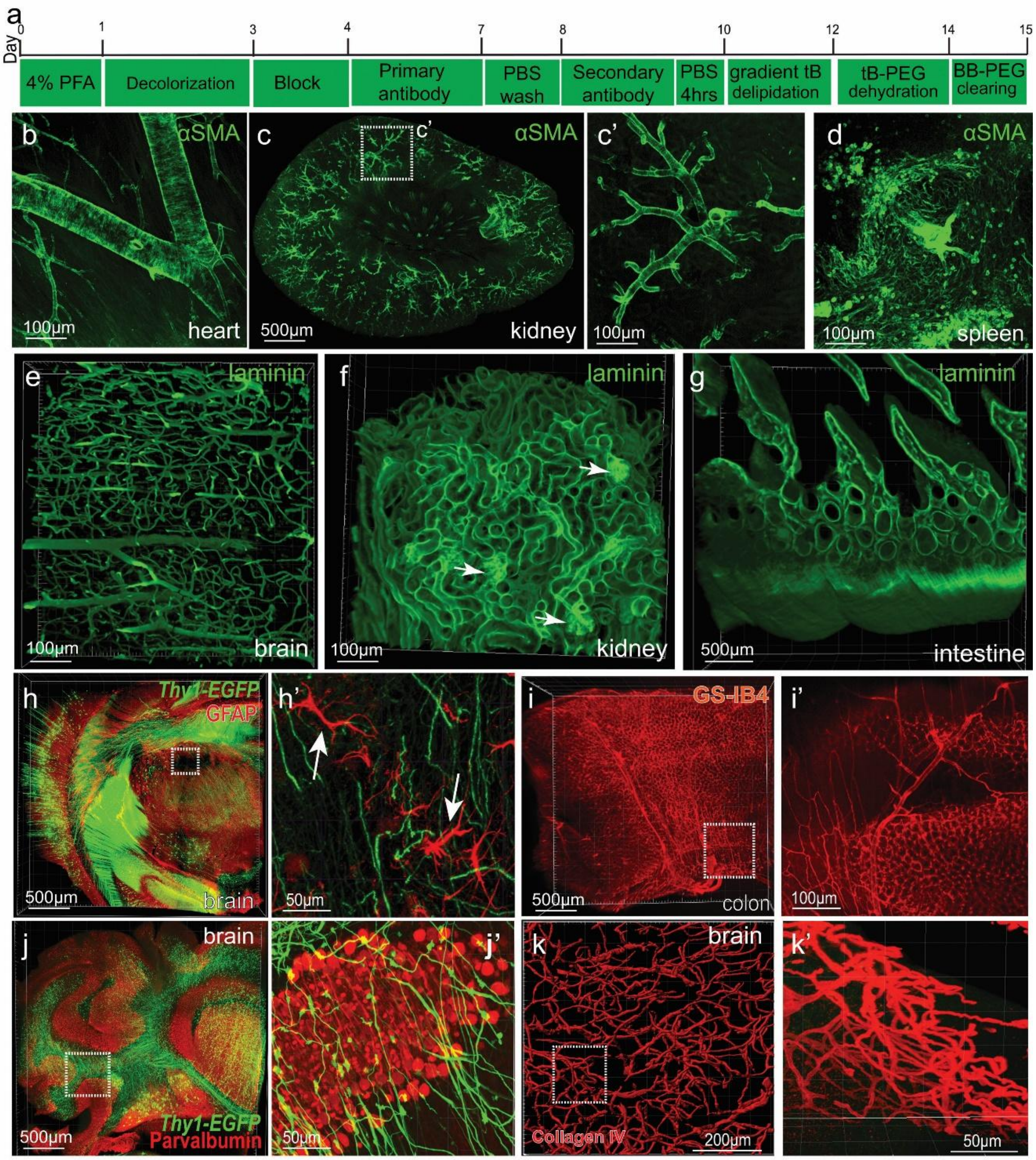
a
Day
0
1
3
4
7
8
10
12
14
15
4% PFA
Decolorization
Block
Primary antibody
PBS wash
Secondary antibody
PBS 4hrs
gradient tB delipidation
tB-PEG dehydration
BB-PEG clearing
b
αSMA
100μm
heart
c
c'
αSMA
500μm
kidney
c'
100μm
d
αSMA
100μm
spleen
e
laminin
100μm
brain
f
laminin
100μm
kidney
g
laminin
500μm
intestine
h
Thy1-EGFP
GFAP
500μm
brain
h'
50μm
i
GS-IB4
500μm
colon
i'
100μm
j
brain
500μm
Thy1-EGFP
Parvalbumin
j'
50μm
k
brain
Collagen IV
200μm
k'
50μm

Figure S7. PEGASOS method is compatible with immunofluorescent, GS-IB4 and EdU staining. (a). Brief description of the passive immersion procedure for whole mount tissue staining. **(b-d)**. Whole-mount immunofluorescent staining using anti-αSMA primary antibody was performed for heart, kidney and spleen slices of 1.5mm thickness. Samples were then cleared following the PEGASOS passive immersion procedure and imaged with a confocal microscope. Boxed area in **(c)** is enlarged in panel **(c')**. **(e-g)**. Whole mount immunofluorescent staining using anti-laminin antibody was performed for brain slices **(e)**, kidney slices **(f)** and intestine slices **(g)** of 1.5mm thickness. Arrows in **(f)** show the renal capsules. **(h)**. Brain slice of 1mm thickness harvested from *Thy1-EGFP* mice was stained with anti-GFAP antibody. Boxed area was enlarged in **(h').** Arrows indicate astrocytes. **(i)**. Colon samples were stained for GS-IB4 and then imaged after clearing. Boxed area is enlarged in **(i')**. **(j)**. Brain slice of 1mm thickness harvested from *Thy1-EGFP* mice was stained with parvalbumin antibody. Boxed area is enlarged in **(j')**. **(k)**. Brain slice of 1mm thickness was stained with anti-collagen IV antibody. Boxed area is enlarged in **(k')**.
