# Supplementary material for: Tissue clearing of both hard and soft tissue organs with the PEGASOS method
Source: Cell Res. 2018 May 29;28(8):803–18. doi: 10.1038/s41422-018-0049-z (PMC6082844; doi:10.1038/s41422-018-0049-z)
Supplement: Supplementary file 17 — Supplementary information, Figure S8 [file 41422_2018_49_MOESM17_ESM.pdf]

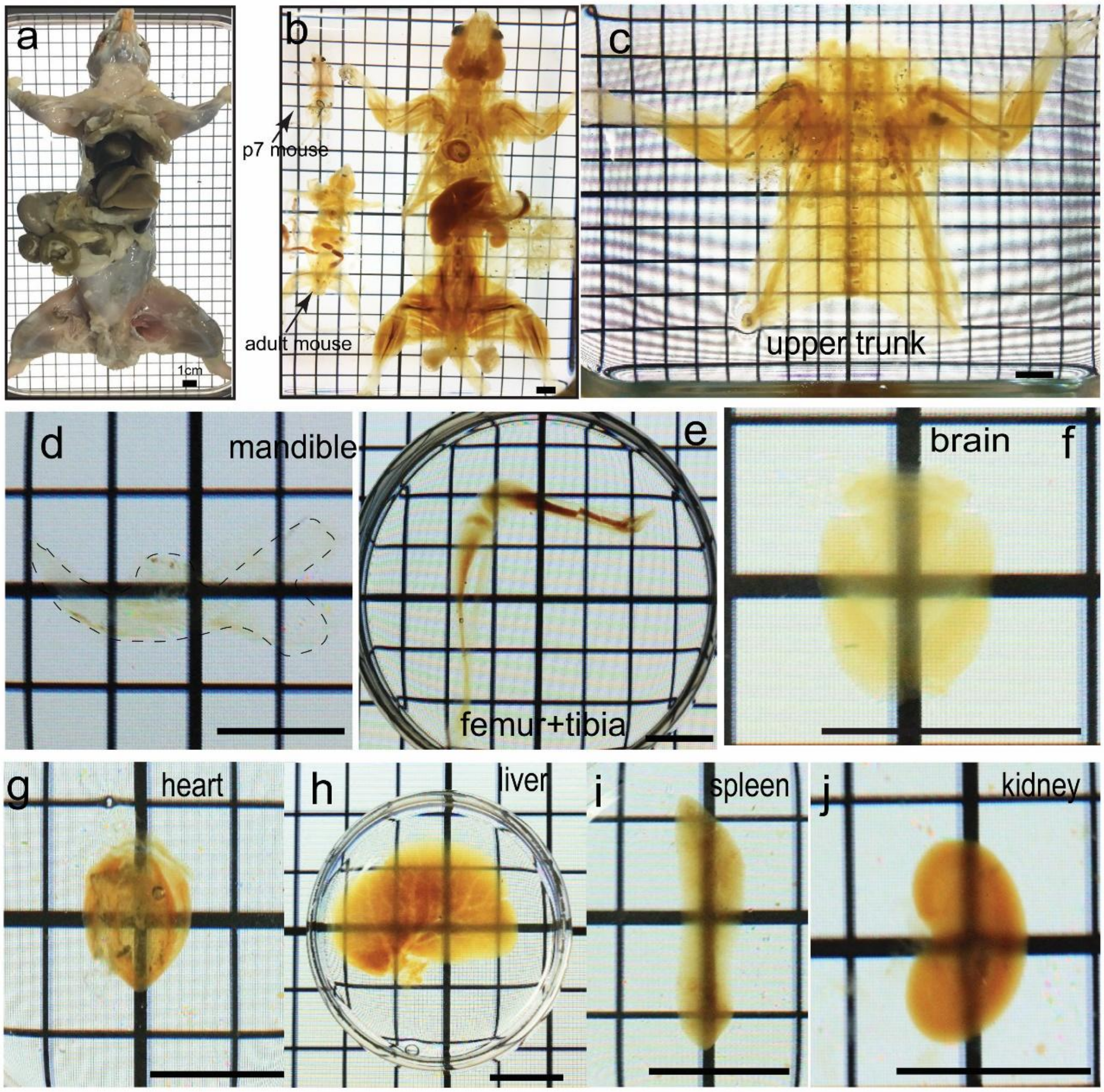
a
1cm
b
p7 mouse
adult mouse
c
upper trunk
d
mandible
e
femur+tibia
brain
f
g
heart
h
liver
i
spleen
j
kidney

Figure S8. PEGASOS recirculation procedure can be scaled up for clearing adult rat. (a, b). Adult rat of 12 weeks age before **(a)** and after **(b)** clearing following the PEGASOS recirculation procedure. An adult (6 weeks age) mouse and a pup (p7) mouse were placed on the left as the size controls after clearing. **(c-j)**. Body parts and organs were dissected and imaged. Dotted lines outline the nearly invisible mandible within the BB-PEG clearing medium. Scale Bars, 1cm.
