# Supplementary material for: Tissue clearing of both hard and soft tissue organs with the PEGASOS method
Source: Cell Res. 2018 May 29;28(8):803–18. doi: 10.1038/s41422-018-0049-z (PMC6082844; doi:10.1038/s41422-018-0049-z)
Supplement: Supplementary file 18 — Supplementary information, Figure S9 [file 41422_2018_49_MOESM18_ESM.pdf]

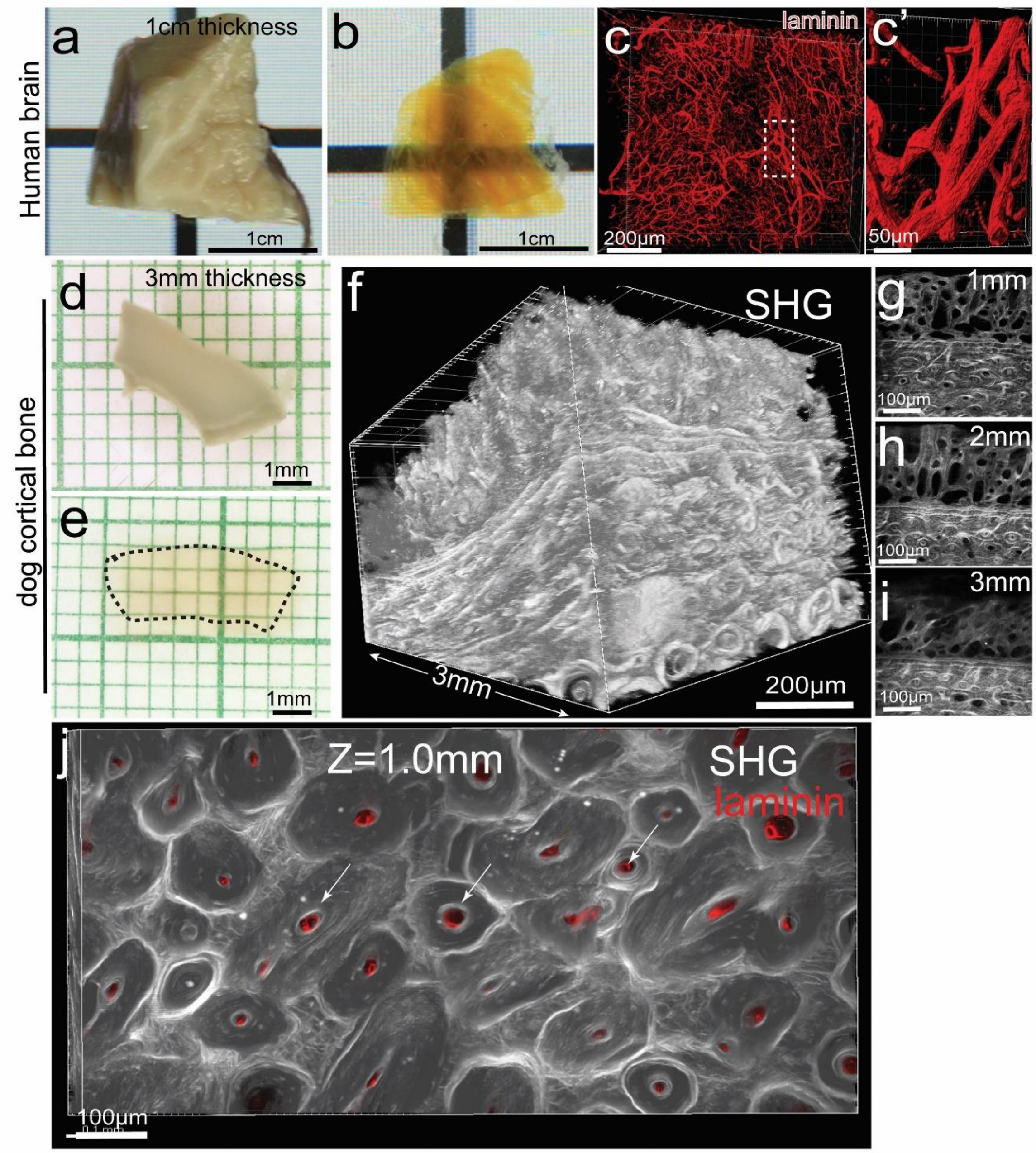
Human brain
a
1cm thickness
1cm
b
1cm
c
laminin
200µm
c'
50µm
dog cortical bone
d
3mm thickness
1mm
e
1mm
f
SHG
3mm
200µm
g
1mm
100µm
h
2mm
100µm
i
3mm
100µm
j
Z=1.0mm
SHG
laminin
100µm

Figure S9. The PEGASOS passive immersion procedure can be scaled up for clearing large tissue samples including human brain and dog bone. **(a).** Fixed human brain sample was cleared following the passive immersion procedure and achieved transparency **(b)**. **(c)**. A slice of human brain sample (1mm thickness) was stained with anti-laminin antibody and imaged after clearing. Boxed region is enlarged in **c'** to show the vascular organization. **(d).** A bone piece (7mm X 3mm X 3mm) harvested from dog tibia was cleared to achieve transparency **(e)**. Cleared dog bone was imaged with a two-photon microscope and an image stack is displayed. Optical sections acquired at different depth are shown in panels (**g)**, (**h)** and **(i)**. **(j).** The anti-laminin antibody staining followed by clearing reveals the Haversian Canals (arrows) located in the center of the osteon. SHG, Second harmonic generation signal.
