# Supplementary material for: Tissue clearing of both hard and soft tissue organs with the PEGASOS method
Source: Cell Res. 2018 May 29;28(8):803–18. doi: 10.1038/s41422-018-0049-z (PMC6082844; doi:10.1038/s41422-018-0049-z)
Supplement: Supplementary file 19 — Supplementary information, Figure S10 [file 41422_2018_49_MOESM19_ESM.pdf]

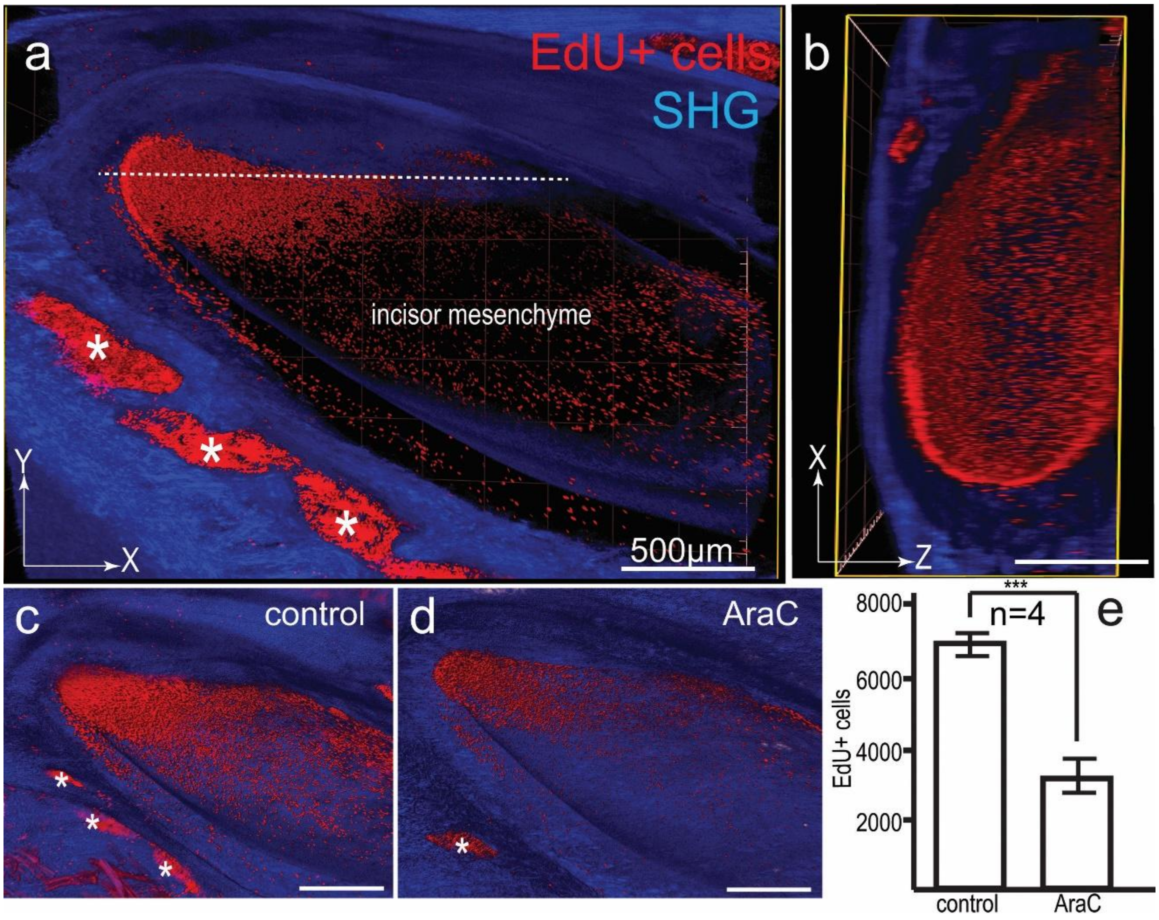
a
EdU+ cells
SHG
incisor mesenchyme
Y
X
500µm
b
X
Z
c
control
d
AraC
e
***
n=4
8000
6000
4000
2000
EdU+ cells
control
AraC

Figure S10. Cytosine arabinoside (Ara-C) treatment reduced the number of transit amplifier cells (TA cells) within the mouse incisor. EdU was injected to adult mice two hours before sacrifice. The mandibles were processed for whole mount EdU staining and clearing. Samples were imaged with a 10X objective on a two-photon microscope. **(a)**. XY stack was acquired to display the EdU+ cells within the incisor mesenchyme and bone marrow (asterisks). **(b).** XZ slice was acquired. The location was indicated with dotted line in **(a)**. Adult mice were injected with either PBS (control) or AraC for two weeks and processed as described above. 3-D images were acquired for control incisor **(c)** and AraC treated incisor **(d)**. Results were quantified in (**e**). All values are mean ± s.d., SHG, Second harmonic generation signal. Statistical significance (***, $P < 0.001$) was assessed by Student *t-test***.** Scale bars, 500µm.
