# Supplementary material for: Tissue clearing of both hard and soft tissue organs with the PEGASOS method
Source: Cell Res. 2018 May 29;28(8):803–18. doi: 10.1038/s41422-018-0049-z (PMC6082844; doi:10.1038/s41422-018-0049-z)
Supplement: Supplementary file 20 — Supplementary information, Table S1 [file 41422_2018_49_MOESM20_ESM.pdf]

Table S1. PEGASOS immersion method time schedule.

		Soft tissue organs	Hard tissue	Tissue slices	
decalcification	20% EDTA	none	4 days with daily change	none	37°C in the shaker
decolorization	25% Quadrol	2 days with daily change	2 days with daily change	1 day	
	Ammonium solution	6 hours for heavily colorized organs	6 hours for heavily colorized long bone	none	
delipidation	30% tert-butanol	4 hours	4 hours	2 hours	
	50% tert-butanol	6 hours	6 hours	4 hours	
	70% tert-butanol	1 days	1 days	4 hours	
dehydration	tB-PEG	2 days with daily change	2 days with daily change	1 day	
Clearing	BB-PEG	1 day	1 day	0.5 day	
Total time		6-7 days	11-12 days	3-4 days	
