# Supplementary material for: Tissue clearing of both hard and soft tissue organs with the PEGASOS method
Source: Cell Res. 2018 May 29;28(8):803–18. doi: 10.1038/s41422-018-0049-z (PMC6082844; doi:10.1038/s41422-018-0049-z)
Supplement: Supplementary file 21 — Supplementary information, Data S1 [file 41422_2018_49_MOESM21_ESM.pdf]

Supplementary information, Data S1.

PEGASOS passive immersion protocol for clearing adult mice organs

Materials:

Quadrol; Sigma-Aldrich; 122262
Tert-Butanol; Sigma-Aldrich; 471712
PEG-MMA500; Sigma-Aldrich; 447943
Benzyl Benzoate; Sigma-Aldrich; B6630
EDTA (Ethylenediaminetetraacetic acid disodium salt dehydrate); Sigma-Aldrich; E5134
Glass vials or 50ml conical tubes

Solutions:

0.5M EDTA (pH=7.0-7.5)
25% w/v Quadrol solution (Quadrol is very vicious and weighing is easier)
3% ammonium solution
30% v/v tert-Butanol + 3% w/v Quadrol
50% v/v tert-Butanol + 3% w/v Quadrol
70% v/v tert-Butanol + 3% w/v Quadrol
[70% v/v tert-Butanol + 30% v/v PEG-MMA-500] + 3% w/v Quadrol
BB-PEG clearing solution (**R.I.** 1.543): [75% v/v Benzyl Benzoate + 25% v/v PEG-MMA-500] + 3% w/v Quadrol

Procedures:

Samples can be placed in 50ml conical tubes for treatment in each step.

1. Perfusion to sacrifice mice and harvest samples: transcardiac perfuse with PBS and then 4% PFA to wash away as much blood as possible.
   The indication of a successful transcardiac perfusion is the liver turning pale.
2. Fixation: fix samples with 4%PFA at 4 ℃ overnight.
3. Decalcification: (for hard tissue only)
   Immerse samples in 0.5M EDTA and place in 37 ℃ shaker (~100rpm) for 2-4 days with daily medium change. The duration of decalcification depends on the size of the bone. Usually adult mouse femur or mandible requires 4 days for complete decalcification.
4. Decolorization:
   --Place whole organs into 25% Quadrol for 1-2 days at 37 ℃ under constant shaking (~100 rpm).
   --Refresh medium daily.
   --(optional) 3% ammonium solution 6 hours for heavily colorized samples that were not perfused.
5. (Optional) Whole mount immunofluorescent staining of samples.
6. Delipidation and dehydration will be performed in 37 ℃ shaker (~100rpm). See table below for recommended duration for each step.
   a. 30% tert-Butanol + 3% Quardol
   b. 50% tert-Butanol + 3% Quardol
   c. 70% tert-Butanol + 3% Quardol

   d. [70% tert-Butanol + 30% PEG-MMA-500]+ 3% Quardol

7. Clearing:
   Briefly rinse the sample with the BB-PEG clearing medium and then immerse and shake it at 37 ℃ (~100rpm) until transparency is reached.
   Tip: Freshly made BB-PEG solution is colorless and turns slightly yellowish after exposing in air. Somehow, we noticed BB-PEG exposed in air for 12 hours achieved better transparency than fresh BB-PEG.

Optimal duration for processing samples of different tissue types and size should be determined by experiments. The following table provides recommended time for different samples.

		Soft tissue organs (adult brain)	Hard tissue (adult mouse femur)	Tissue Slices (brain slice of 1-2mm thickness)	
decalcification	0.5M EDTA	none	4 days with daily change	none	37 ℃ in the shaker
decolorization	25% Quadrol	2 days with daily change	2 days with daily change	1 day	
	Ammonium solution	6 hours for heavily colorized organs	6 hours for heavily colorized long bone	none	
delipidation	30% tert-butanol	4 hours	4 hours	2 hours	
	50% tert-butanol	6 hours	6 hours	4 hours	
	70% tert-butanol	1 days	1 days	4 hours	
dehydration	tB-PEG	2 days with daily change	2 days with daily change	1 day	
Clearing	BB-PEG	1 day	1 day	0.5 day	
Total time		6-7 days	11-12 days	3-4 days	

8. Storage: Samples can then be preserved in the clearing medium covered with aluminum foil at room temperature for a long term.
