# Supplementary material for: Tissue clearing of both hard and soft tissue organs with the PEGASOS method
Source: Cell Res. 2018 May 29;28(8):803–18. doi: 10.1038/s41422-018-0049-z (PMC6082844; doi:10.1038/s41422-018-0049-z)
Supplement: Supplementary file 22 — Supplementary information, Figure S1 [file 41422_2018_49_MOESM22_ESM.pdf]

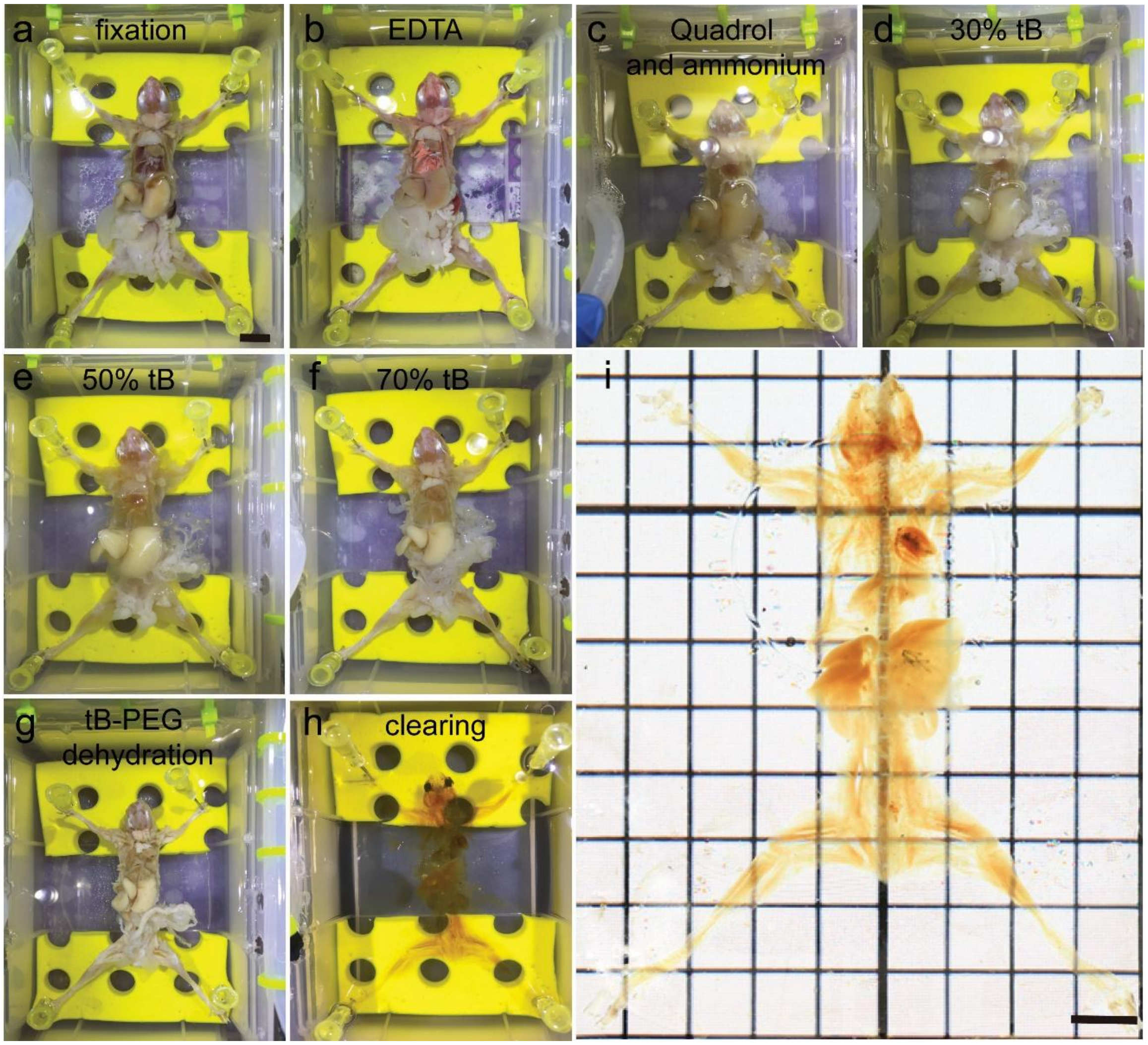
a
fixation
b
EDTA
c
Quadrol
and ammonium
d
30% tB
e
50% tB
f
70% tB
i
g
tB-PEG
dehydration
h
clearing

Figure S1. Clearing of adult whole mouse following the PEGASOS full body recirculation procedure. *C57BL/6* mouse of 2 months age was processed following the full body recirculation procedure. Images were acquired after fixation (a), EDTA decalcification (b), quadrol and ammonium decolorization (c), de-lipidation with 30% tB (d), 50% tB (e) and 70% tB (f), dehydration with tB-PEG (g) and clearing (h). Eyeballs were removed and the mouse was placed on illuminated background for imaging (i). Scale bars, 1cm.
