# Supplementary material for: Tissue clearing of both hard and soft tissue organs with the PEGASOS method
Source: Cell Res. 2018 May 29;28(8):803–18. doi: 10.1038/s41422-018-0049-z (PMC6082844; doi:10.1038/s41422-018-0049-z)
Supplement: Supplementary file 23 — Supplementary video legend [file 41422_2018_49_MOESM23_ESM.docx]

**Titles of supplementary videos**

**Video 1.** 3D reconstruction of a *Tie2-Cre;Ai14* mouse head of p7 age.

**Video 2.** Reconstruction of the whole mouse mandible with a 10X objective

**Video 3.** Imaging of an intact femur with a 10X objective

**Video 4.** Imaging of a *Thy1-EGFP* mouse brain with a 10X objective.

**Video 5.** Tracing of individual neuron and axon within adult brain of *Thy1-EGFP* mouse with 20X objective

**Video 6.** Whole brain vasculature imaging of a *TTH* mouse brain with a tiling light sheet microscope.

**Video 7.** Cervical spinal cord and DRGs within the vertebrae of a *Thy1-EGFP* mouse imaged with a tiling light sheet microscope.

**Video 8.** Tracing of DRG central axon branches with a 10X objective on a confocal microscope.

**Video 9.** Tracing of individual neurons and axons within a DRG of adult *Thy1-EGFP* mouse imaged with a 20X objective.

**Video 10.** Neural network within the bone marrow space of an adult *Wnt1-Cre;Ai14* mouse tibia imaged with a 20X objective.
